# Exploring the Anti-Inflammatory Potential of *Daucus carota* L. subsp. *carota* Seed Extracts: Phytochemical Profiling, In Vitro Antioxidant Activity and Modulation of the Arachidonic Acid Cascade

**DOI:** 10.3390/plants15152400

**Published:** 2026-08-05

**Authors:** Monica Maio, Gilda D’Urso, Alessandra Capuano, Francesca Fantasma, Michela Aliberti, Ester Colarusso, Gabriella Saviano, Vincenzo De Felice, Paola Fortini, Gianluigi Lauro, Maria Giovanna Chini, Agostino Casapullo, Giuseppe Bifulco, Maria Iorizzi

**Affiliations:** 1 Department of Biosciences and Territory, University of Molise, Contrada Fonte Lappone, 86090 Isernia, Italy; m.maio3@studenti.unimol.it (M.M.); fantasma@unimol.it (F.F.); saviano@unimol.it (G.S.); defelice@unimol.it (V.D.F.); fortini@unimol.it (P.F.); iorizzi@unimol.it (M.I.); 2 Department of Pharmacy, University of Salerno, Via Giovanni Paolo II 132, 84084 Salerno, Italy; gidurso@unisa.it (G.D.); acapuano@unisa.it (A.C.); mialiberti@unisa.it (M.A.); ecolarusso@unisa.it (E.C.); glauro@unisa.it (G.L.); casapullo@unisa.it (A.C.)

**Keywords:** *Daucus carota* L. subsp. *carota*, anti-inflammatory assay, mineral composition, antioxidant assay, LC-MS, qualitative phytochemical analysis

## Abstract

Wild plant biodiversity represents a largely untapped source of chemically diverse metabolites. However, many wild edible species remain poorly characterized despite their potential as sources of antioxidant and anti-inflammatory phytochemicals. *Daucus carota* L. subsp. *carota*, commonly known as wild carrot, has attracted interest due to its long history of medicinal use. In this study, seed extracts collected from populations growing in two Italian regions (Lazio and Molise) were investigated to evaluate their phytochemical composition, mineral content, antioxidant activity, and anti-inflammatory potential. The metabolite profiles were characterized by qualitative Liquid Chromatography-Mass Spectrometry analysis, allowing the identification of several phenolic compounds and other secondary metabolites. Antioxidant activity was assessed using in vitro assays, while anti-inflammatory activity was evaluated through the inhibition of cyclooxygenase and soluble epoxide hydrolase, two key enzymes involved in the arachidonic acid cascade. To gain further insight into the possible mechanisms underlying these activities, molecular docking analyses were performed on selected metabolites identified in the extracts, exploring their interactions with the target enzymes. The extracts displayed promising antioxidant and anti-inflammatory properties, while docking results supported the potential role of specific metabolites in enzyme modulation. These findings highlight the value of underexplored wild *D. carota* seeds as a source of natural compounds with potential applications in the development of nutraceutical and cosmeceutical products.

## 1. Introduction

Wild plants used in traditional medicine constitute a valuable reservoir of bioactive phytochemicals with relevant biological activities, including antioxidant, anti-microbial, and anti-inflammatory properties [1,2], making them attractive candidates for identifying new therapeutic leads for several pathological conditions, particularly inflammation-associated disorders [3,4].

Inflammation is a natural process that plays a crucial role in tissue protection and repair [4,5,6]. It constitutes the body’s defensive response, eliminating harmful agents or limiting their spread by removing damaged cells and tissues or pathogens. Upon exposure to injurious agents, cells activate inflammatory signaling pathways that lead to the production of pro-inflammatory cytokines, chemokines, and other signaling molecules [7,8].

Although acute inflammation is essential for survival, its dysregulation or persistence leads to chronic inflammatory states that contribute to the onset and progression of several pathological conditions, including cancer, cardiovascular diseases, metabolic disorders such as type 2 diabetes, neurodegenerative diseases, and autoimmune disorders [3,4,9]. At the molecular level, inflammatory responses are largely mediated by lipid-derived signaling molecules originating from the arachidonic acid cascade, a central metabolic pathway regulated by cyclooxygenase (COX), lipoxygenase (LO), and cytochrome P450 (CYP) enzymes [10], resulting in the formation of various inflammatory mediators such as prostaglandins, prostacyclin, lipoxins, and leukotrienes [10,11].

Arachidonic acid (AA) metabolism depends on the activity of ω-hydroxylase and epoxigenase, which generate AA epoxides, or eicosatrienoic acid epoxides (EETs). The conversion of EETs into the corresponding diols, or dihydroxy eicosatrienoic acids (DHETs), is primarily catalyzed by soluble epoxide hydrolase (sEH) [12].

These enzymatic systems generate a wide range of bioactive lipid mediators that finely regulate pro- and anti-inflammatory processes, making them key pharmacological targets for potential therapeutic intervention. Among currently available treatments, non-steroidal anti-inflammatory drugs (NSAIDs) primarily inhibit COX enzymes [13], thereby reducing prostaglandin synthesis, whereas corticosteroids (glucocorticoids) inhibit phospholipase A2, limiting the release of arachidonic acid and downstream inflammatory mediators [14]. Despite their clinical efficacy, long-term use of these drugs is often associated with significant adverse effects [15].

Currently, sEH inhibitors are promising pharmacological candidates for a range of human diseases. In preclinical animal models, several sEH inhibitors, including adamantane-1-yl-ureido]-dodecanoic acid (AUDA), 12-(3-Adamantan-1-yl-ureido)dodecanoic acid butyl ester (AUDA-BE), trans-4-[4-(3-Adamantan-1-yl-ureido)cyclohexyloxy]benzoic acid (t-AUCB), 1-(1-Propanoylpiperidin-4-yl)-3-[4-(trifluoromethoxy)phenyl]urea (TPPU), and 1-adamantan-1-yl-3-urea (AEPU), have been shown to reduce multi-organ inflammation effectively [10,16].

Despite the availability of effective anti-inflammatory drugs, developing safer alternatives remains challenging, prompting renewed interest in natural products long used in traditional medicine across cultures to treat inflammatory conditions [17,18].

Wild carrot (*Daucus carota* L. subsp. *carota*) belongs to the Apiaceae (Umbelliferae) family, a group of aromatic flowering plants that includes both edible and toxic species [19]. The genus *Daucus* is widely distributed throughout Europe, Asia, the Americas, and Australia, and is characterized by considerable morphological diversity. Wild carrot is one of the most important taproot crops worldwide, valued for its nutritional composition, attractive coloration, and distinctive taste [20,21]. Carrot taproots are particularly rich in bioactive compounds, including vitamins, β-carotene, sugars, flavonoids, polyacetylenes, phenolic acids, anthocyanins, and dietary fiber [22]. Owing to this diverse phytochemical profile, fresh carrots are extensively consumed as food and are increasingly utilized as raw materials in the nutraceutical, cosmetic, and functional food industries [23].

Depending on pigmentation, carrots are generally classified into Western (orange, yellow, white) and Eastern (purple, red, black) types, mainly due to differences in carotenoid and anthocyanin content [23,24]. Many ethnopharmacological uses of *Daucus carota* have been reported, including its use in traditional medicine and as a food flavoring. In folk medicine, the plant is known for its diuretic and carminative properties, antipyretic, analgesic, anti-inflammatory, anti-diabetic, antilithic, and antiseptic effects [25]. Taproots have good antioxidant, antimutagenic, and immunostimulant properties and anticancer effects [26], while leaves, flowers, and fruits show good anti-microbial activity [20].

Although carrot taproots are the most widely used and representative part in folk medicine, seeds have long been used in Ayurvedic and European herbal medicine for their diuretic, carminative, and detoxifying properties. The wild species is protected under regional biodiversity conservation projects in Italy, such as those in Sardinia (Regional Law No. 16 of 07/08/2014) and Apulia (BiodiverSO Project), and is recognised as a native species and a key component of coastal and grassland ecosystems [19]. The fruits of the carrot are schizocarps, which are dry fruits that, upon maturity, split into two single-seeded units known as mericarps. The essential oil extracted from the seeds is widely used in the food flavoring, cosmetics, and perfumery industries and has been reported to exhibit anti-microbial, hepatoprotective, and tonic properties [27,28,29]. The main chemical constituents of the different chemotypes of seed essential oil include α-pinene, β-bisabolene, β-pinene, γ-terpinene, camphene, carotol, geranyl acetate, limonene, myrcene, sabinene, and daucol [27,28]. Despite the increasing interest in *D. carota*, limited studies have focused on the chemical characterization and anti-inflammatory potential of polar seed extracts, particularly those derived from different geographical origins [25,30]. Comprehensive metabolomic profiling of seed extracts combined with multi-target evaluation of their anti-inflammatory activity remains largely unexplored. This study aimed to characterize the mineral and phytochemical composition of seed extracts from wild *Daucus carota* L. subsp. *carota* and to evaluate their antioxidant and anti-inflammatory potential. To achieve these objectives, the extracts were fractionated and chemically characterized by LC-MS. In parallel, antioxidant activity was assessed through in vitro assays. Based on the outcomes of these preliminary investigations, the most relevant fraction was selected for further evaluation of its anti-inflammatory potential through the inhibition of cyclooxygenase-2 (COX-2) and soluble epoxide hydrolase (sEH), key enzymes involved in inflammatory pathways (vide supra). To support the interpretation of biological data, molecular docking studies were performed on selected metabolites identified by LC-MS analysis to investigate their potential interactions with the target enzymes. By integrating metabolomic profiling, bioactivity assessment, and in silico analysis, this study sought to identify metabolites potentially associated with the observed biological effects and to provide new insights into the valorization of wild *D. carota* L. subsp. *carota* seeds as sources of bioactive compounds for use in the development of nutraceutical and cosmeceutical products, or as natural preservative agents, e.g., in the food or cosmetic industries, to extend shelf life.

## 2. Results and Discussion

**Figure 1:**
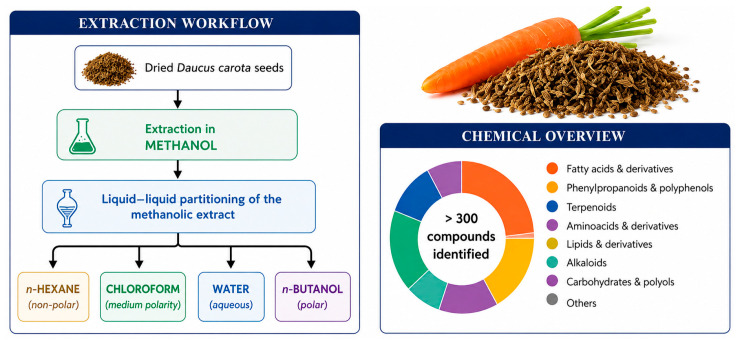
Schematic workflow for the study of *D. carota* subsp. *carota* seeds.

The biological potential of carrot seeds from two different geographical origins, DCC (*D. carota* L., Capracotta) and DCA (*D. carota* L., Amatrice), was investigated through an integrated analytical approach (Figure 1). The obtained results are presented and discussed according to the sequence of analyses performed, including in vitro antioxidant assays, elemental profiling, phytochemical characterization, anti-inflammatory activity evaluation, and molecular docking studies.

### 2.1. Total Phenolic Content (TPC) and Total Flavonoid Content (TFC)

As shown in Table 1, TPC and TFC values were unevenly distributed among the extracts and fractions.

The *n*-BuOH fraction exhibited the highest TPC values in both accessions, with 38.49 ± 1.31 mg GAE/g DE for DCC and 32.18 ± 1.57 mg GAE/g DE for DCA. TFC distribution differed between the two accessions: the highest value in DCC was observed in the *n*-BuOH fraction, whereas the highest value in DCA was found in the 80% MeOH extract. The aqueous fraction showed the lowest TPC and TFC values.

This distribution is consistent with previous reports indicating that the solvent polarity is a key factor in the extraction of phenolic compounds. In particular, polar solvents such as methanol and water are particularly suitable for polyphenol extraction, whilst non-polar solvents tend to be less effective [25].

Moreover, solvents with intermediate polarity facilitate the extraction of specific classes of phenolic compounds and flavonoids, found in higher concentrations in the semi-polar fractions [20].

The total phenolic and flavonoid contents were not determined in the non-polar fractions (*n*-hexane and chloroform) due to the high concentration of lipid compounds, which caused interference in the spectrophotometric assays, thus compromising the reliability of the results. Two-way ANOVA showed that solvent fraction significantly affected both TPC and TFC (*p* < 0.0001). A significant population × solvent interaction was also observed for both variables (*p* < 0.0001), indicating that differences between DCC and DCA depended on the solvent fraction considered. The main effect of population was not significant for TPC (*F* = 0.1, *p* = 0.756), whereas it was significant for TFC (*F* = 17.3, *p* = 0.0013). Because significant interactions were detected, differences among individual population–solvent combinations were evaluated using Tukey’s HSD post hoc test, as indicated by the superscript letters in Table 1.

### 2.2. Antioxidant Capacity

As reported in Table 2, antioxidant capacity measured by the DPPH and ABTS assays differed markedly among the extracts and fractions. In both DCC and DCA, the *n*-BuOH fraction showed the highest antioxidant activity, with values ranging from 64.00 to 88.04 mg TE/g extract for DPPH and from 80.08 to 118.02 mg TE/g extract for ABTS. Intermediate values were observed for the 80% MeOH extract, whereas the aqueous fraction showed low DPPH activity but retained appreciable ABTS scavenging capacity. Remarkably low DPPH values were observed for the non-polar *n*-hexane fraction, ranging from 0.28 to 0.38 mg TE/g extract.

The differences observed between the DPPH and ABTS results may be related to the distinct chemical characteristics and reaction conditions of the two assays. The ABTS radical cation can react with both hydrophilic and lipophilic antioxidants and is applicable in aqueous as well as organic media. In contrast, the DPPH assay is generally performed in organic media and may be less responsive to highly polar antioxidants or compounds characterized by slower reaction kinetics. These methodological differences may explain the markedly higher ABTS activity observed for the aqueous fractions compared with the corresponding DPPH values. This finding suggests that the aqueous fractions may contain hydrophilic antioxidant constituents other than phenolic compounds, including organic acids and other small polar metabolites. Conversely, the high activity of the *n*-BuOH fractions in both assays agrees with their enrichment in phenolic, flavonoid, and other redox-active compounds able to react efficiently with both radicals. Overall, these results confirm that solvent polarity strongly influences the recovery of antioxidant constituents from the extract [20].

Complementary pairwise Student’s *t*-tests were used to compare DCC and DCA within each solvent fraction. For DPPH, significant differences that remained significant after Holm correction were observed for *n*-BuOH, 80% MeOH, and CHCl_3_. The *n*-BuOH fraction showed higher activity in DCC than in DCA (88.04 ± 1.50 vs. 64.00 ± 0.82 mg TE/g extract; adjusted *p* = 0.000084), whereas the 80% MeOH extract (14.78 ± 0.60 vs. 17.08 ± 0.13 mg TE/g extract; adjusted *p* = 0.0120) and CHCl_3_ fraction (9.93 ± 0.25 vs. 11.50 ± 0.47 mg TE/g extract; adjusted *p* = 0.0205) showed higher activity in DCA. No significant differences were found for the aqueous or n-hexane fractions.

For ABTS, nominal differences were observed for *n*-BuOH, with higher activity in DCC, and for 80% MeOH, with higher activity in DCA. However, neither comparison remained significant after Holm correction. These results indicate that population-related differences were more robust for DPPH than for ABTS and were strongly dependent on the solvent fraction, in agreement with the significant population × solvent interaction.

Two-way ANOVA showed that solvent fraction significantly affected both DPPH and ABTS antioxidant activity (*p* < 0.0001). A significant population × solvent interaction was also observed for both assays (*p* < 0.0001), demonstrating that differences between DCC and DCA depended on the solvent fraction considered. The main effect of population was significant for both DPPH (*F* = 313.9, *p* < 0.0001) and ABTS (*F* = 24.0, *p* < 0.0001). Because significant interactions were detected, differences among individual population–solvent combinations were evaluated using Tukey’s HSD post hoc test, as indicated by the superscript letters in Table 2.

### 2.3. Elemental Profiles

As shown in Table 3, after adjustment for multiple comparisons, the concentrations of B, Ba, Fe, K, Mg, Mn, Ni, and P differed significantly between DCA and DCC, whereas no significant differences were observed for Cu, S, and Zn. These differences can be attributed to variations in soil and climate conditions between the two study areas. Several environmental factors have been reported to influence the uptake of mineral nutrients by plants, including temperature, seasonality, and CO_2_ levels. In addition, plant-related factors such as genetics, variety, root architecture, and growth stage may also play a role in this process [31]. In both samples, K was the most abundant element, with concentrations ranging from 4853.89 to 7573.33 mg kg^−1^ DM, followed by P (4747.58–6498.01 mg kg^−1^ DM), Mg (2406.19–2894.46 mg kg^−1^ DM), and S (2178.00–2338.38 mg kg^−1^ DM). Compared with the DCC sample, the DCA sample had higher concentrations of most of the analysed elements, such as B, Ba, K, Mg, Ni, P, S, and Zn. Conversely, DCC showed higher concentrations of Fe, Cu, and Mn than DCA. The contents of the microelements Ni, Cu, and Zn were in line with those reported by Hadini et al., namely 3.12, 18.58, and 70.01 mg kg^−1^ DM, respectively [32].

### 2.4. Phytochemical Profiles of Two Daucus carota L. Seeds by LC-MS Analysis

The phytochemical profiles of two distinct *Daucus carota* accessions, DCA and DCC, were investigated using a two-step analytical workflow. Initially, a hydroalcoholic extraction was performed using 80% methanol to obtain a broad representation of polar and semi-polar constituents (Supplementary Material Figure S1).

To achieve more targeted fractionation based on chemical polarity, a modified Kupchan partitioning procedure was subsequently applied to a methanolic extract, yielding four distinct fractions: *n*-hexane, chloroform, *n*-BuOH, and water.

All extracts and fractions were qualitatively characterized by High-Performance Liquid Chromatography coupled to tandem High-Resolution Mass Spectrometry (LC-ESI-MS/MS) on a Q-Exactive Orbitrap mass spectrometer in positive and negative ion modes (LC-MS profiles are reported in the Supplementary file, Figures S2–S9). This high-resolution platform enabled the putative identification and profiling of specialized metabolites discussed below.

#### 2.4.1. Untargeted Metabolomic Profiling of the Hydroalcoholic Extracts by LC-MS and Molecular Networking

**Figure 2:**
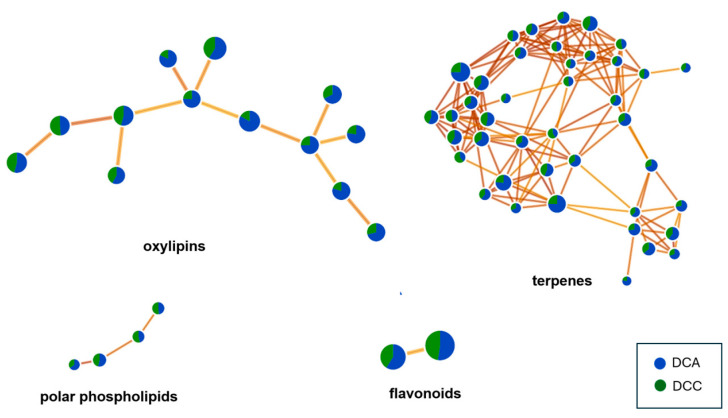
MS/MS-based molecular networking of DCA and DCC hydroalcoholic (80% methanol) extracts generated via Compound Discoverer. Nodes represent individual precursor ions, whereas edges connect features sharing MS/MS spectral similarity. Pie charts within nodes indicate the relative peak area contribution of each compound in DCA (blue) and DCC (green). Four major chemical families were identified, including oxylipins, terpenes, polar phospholipid derivatives, and flavonoid-related metabolites. The terpene and oxylipin clusters exhibited the greatest structural expansion and a predominance of DCA-associated nodes, whereas the polar phospholipid and flavonoid clusters displayed a largely conserved distribution between the two accessions.

Untargeted metabolomic profiling of the hydroalcoholic (80% methanol) extracts, conducted via LC-MS and processed in Compound Discoverer, led to the putative identification of several specialized metabolites and several unknown compounds. An initial molecular networking analysis of these extracts was performed using Compound Discoverer, grouping metabolites based on MS/MS spectral similarity, revealing a highly structured chemical space organized into distinct, well-defined clusters that organize detected features into a network based on the similarity of their MS/MS fragmentation spectra. In this approach, each node represents a detected metabolite (or molecular feature), while edges connect nodes sharing similar fragmentation patterns, as quantified by spectral similarity scores. Since structurally related compounds often generate comparable MS/MS spectra, the resulting network groups metabolites into clusters that are likely to represent chemically related molecular families. This analysis revealed a highly structured chemical space composed of distinct, well-defined clusters, facilitating the visualization of relationships among metabolites and the identification of structurally related compounds (Figure 2). Four major chemical families emerged as dominant features of the molecular network: oxylipins, terpenes, polar phospholipid derivatives, and flavonoid-based secondary metabolites. The pie charts associated with each node represent the relative contribution of the two sample matrices (DCA and DCC), with the size of each coloured sector indicating the proportion of ion intensity attributed to each matrix (DCA in blue and DCC in green). Overall, polar phospholipid and flavonoid clusters were characterized by pie charts containing similar proportions of both colors, indicating that these metabolite classes were broadly shared between DCA and DCC. In contrast, the oxylipins, terpene and terpenoid-related clusters displayed a predominance of pie charts enriched in the DCA fraction, together with a larger number of nodes and a more complex network topology, suggesting a higher abundance and structural diversity of terpenes in DCA compared with DCC.

The analysis of these clusters led to the putative annotation of several metabolites based on spectral matches and database comparisons. At the same time, a considerable proportion of nodes remained uncharacterized (unknown features), reflecting the intrinsic chemical complexity of the samples.

In Table 4, the metabolites identified in hydroalcoholic extracts of Amatrice (DCA) and Capracotta (DCC) seeds are reported, revealing a diverse array of metabolites. This solvent proved chemically versatile, retaining not only central metabolic intermediates such as citric acid and quinic acid but also a broad spectrum of amino acids, including arginine, phenylalanine, and tryptophan, along with sugar alcohols and small carbohydrate derivatives.

Notably, the extract also preserved a substantial portion of phenylpropanoid and flavonoid chemistry, including chlorogenic acid, luteolin and its glycosylated forms, umbelliferone, and isoferulic acid, highlighting methanol as an effective solvent for bridging primary metabolism with antioxidant secondary metabolism. In addition, lysophospholipids, sphingoid bases, and oxylipin-related species, such as hydroxy- and epoxy-fatty acid derivatives, were co-extracted.

#### 2.4.2. Analysis of Apolar Fractions Obtained by the Modified Kupchan Partitioning Procedure

In a second analytical step, the crude methanolic extract was subjected to polarity-driven liquid–liquid partitioning into *n*-hexane, chloroform, *n*-BuOH, and aqueous fractions to achieve a deeper resolution of metabolite classes and improve the chemical characterization of the extract. This fractionation strategy enabled progressive enrichment of chemically related metabolites and facilitated more detailed exploration of class-specific metabolic patterns. Across all fractions, the two matrices displayed a largely conserved metabolomic architecture, with differences primarily reflected in relative abundance rather than in the presence or absence of compounds (see Supplementary Tables S1–S5).

In detail, the *n*-hexane fraction (Supplementary Table S4) was characterized by aliphatic and unsaturated fatty acid derivatives (hexadeca-4,7,10,13-tetraenoic acid, oleic acid, azelaic acid), long-chain aldehydes (tridecanal, 2,4-undecadienal), aromatic aliphatic compounds (1-phenyl-2-hexanone, 5-phenyl-1-pentanol), and terpene-related structures, including caryophyllene oxide, camphor, and carvone, most of which are already reported in *D. carota* L. species [25,33]. In addition, the detection of oxylipins such as 12,13-DHOME and 13-oxo-octadecadienoic acid indicates that even highly apolar extraction conditions retain a fraction of oxidized lipid mediators, likely due to their partial hydrophobic character and membrane association. The presence of sphingoid bases (sphinganine, sphingosine, phytosphingosine) and monoacylglycerols further supports the interpretation that this fraction is strongly enriched in membrane-core lipid constituents and their breakdown products.

The intermediate chloroform fraction (Supplementary Table S5) was dominated by terpenoids, sesquiterpenoids, and phenylpropanoid derivatives, confirming the prominence of isoprenoid metabolism observed in the molecular network, with DCA retaining slightly higher chemical diversity within this class. The chloroform fraction was characterized by a complex mixture of lipid-associated metabolites and lipophilic secondary compounds, including terpenoids such as caryophyllene oxide, camphor, and carvone, together with aromatic prenylated structures and phenylpropanoid derivatives (myristicin, isoferulic acid, acetovanillone, demethylsuberosin, isoimperatorin, phellopterin). In parallel, this fraction retains a substantial pool of structural lipids and lipid-derived mediators, including saturated and unsaturated fatty acids (palmitic, stearic, oleic, and linoleic acids), oxidized fatty acids (13-HODE), monoacylglycerols, sphingoid bases (sphingosine, phytosphingosine), and lyso-lipidic species. Notably, despite its overall hydrophobic nature, the chloroform fraction still contains selected polar contaminants such as mannitol and certain flavonoid derivatives. These data reflect the inherent complexity and partial overlap of solvent partitioning in biological matrices.

#### 2.4.3. Analysis of Polar Fractions of DCC and DCA Obtained by the Modified Kupchan Partitioning Procedure

The *n*-BuOH fraction (Supplementary Table S1) enriched polar phenolics, particularly flavonoids and phenylpropanoid acids, which constituted the major pool of antioxidant-related metabolites in both matrices. Among the most representative constituents, oxidized fatty acids and oxylipins (e.g., 13-HODE, 12,13-DHOME, hydroxy-fatty acids, and dicarboxylic acids) were highly abundant, suggesting that the *n*-BuOH fraction efficiently recovered lipid-derived signaling molecules and oxidative metabolites. These compounds are increasingly recognized for their roles in modulating inflammation, membrane remodeling, and stress-response pathways, thereby conferring strong functional significance to the fraction. Meanwhile, enrichment of flavonoids and polyphenolic derivatives, including luteolin-, apigenin-, and kaempferol-related metabolites, together with phenylpropanoids such as myristicin, coniferyl aldehyde, and ferulate derivatives, indicates a substantial concentration of antioxidant and redox-active phytochemicals.

Finally, the aqueous fraction (Supplementary Table S3) contained primary metabolites, including amino acids, organic acids, and sugar derivatives, that were highly conserved across the two origins. Overall, the metabolite distribution demonstrates that the *n*-butanolic partition serves as a highly selective reservoir of bioactive, semi-polar compounds, bridging lipidic and phenolic chemical spaces. This compositional pattern is particularly noteworthy because it reflects *n*-BuOH capacity to enrich metabolites associated with antioxidant, anti-inflammatory, and signaling functions, thereby providing a chemically and pharmacologically valuable fraction for further biological and nutraceutical investigations.

During Kupchan partitioning, a precipitate was recovered from the *n*-BuOH extract and identified as mannitol by ^1^H, ^13^C, and DEPT 135° NMR experiments (see Supplementary Material Figures S10–S17 and Table S6) and LC-MS analysis (Table 4). Mannitol, also known as mannite or manna sugar [(2*R*,3*R*,4*R*,5*R*)-hexane-1,2,3,4,5,6-hexol], is a naturally occurring polyalcohol widely distributed in nature, where it functions as a carbon reserve and osmo-protectant [34,35]. Previously, mannitol had only been identified in carrot roots [36]; to the best of our knowledge, this is the first identification of mannitol in carrot seeds.

Due to its favorable properties, including non-hygroscopicity, high stability, low caloric value, and sweetness, mannitol is widely used in the food, pharmaceutical, and chemical industries [37]. It is also employed in several pharmaceutical applications, including the management of intracranial and intraocular pressure and as an inhaled adjunct therapy for cystic fibrosis [38].

### 2.5. Cell-Free Activity Assays on COX-2 and sEH

Based on the preliminary results of the in vitro antioxidant activity tests and subsequent characterization of the extracts, the *n*-BuOH fractions were selected for an in-depth assessment of their anti-inflammatory potential. This assessment focused on two key enzymes involved in inflammatory pathways: cyclooxygenase-2 (COX-2) and soluble epoxide hydrolase (sEH).

The enrichment of flavonoids, phenylpropanoids, and oxidized fatty acid derivatives suggests the modulation of inflammation-related pathways. These metabolites are commonly associated with anti-inflammatory and antioxidant activities and may contribute to the regulation of immune responses as well as the mitigation of oxidative stress. In view of the previously reported inhibitory activity of carrot seed-derived extracts against COX-2 [39], we first investigated whether the samples in our hands exhibited similar effects on this target.

**Figure 3:**
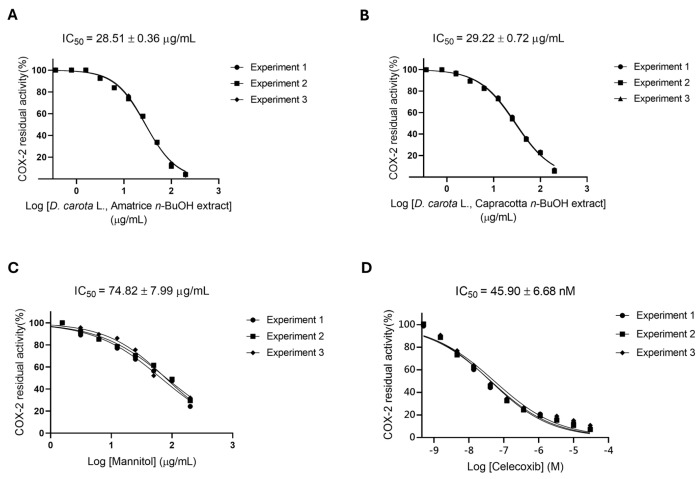
IC_50_ curves of DCA *n*-BuOH (**A**), DCC *n*-BuOH (**B**), mannitol (**C**), and celecoxib (**D**) against COX-2 enzyme; data are expressed as means ± SD, for *n* = 3. DCA: ${\bar{R}}^{2}=0.9949$; DCC: ${\bar{R}}^{2}=0.9958$; Mannitol: ${\bar{R}}^{2}=0.9827$; Celecoxib: ${\bar{R}}^{2}=0.9723.$

**Figure 4:**
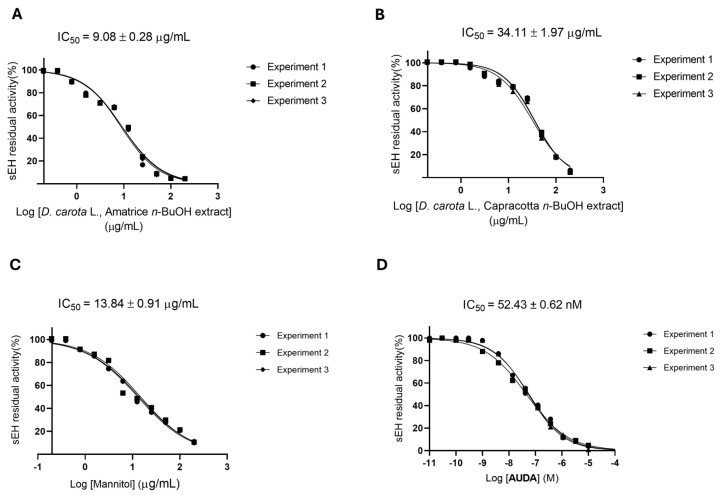
IC_50_ curves of DCA *n*-BuOH (**A**), DCC *n*-BuOH (**B**), mannitol (**C**), and AUDA (**D**) against sEH enzyme; data are expressed as means ± SD, for *n* = 3. DCA: ${\bar{R}}^{2}=0.9827$; DCA: ${\bar{R}}^{2}=0.9844$; Mannitol: ${\bar{R}}^{2}=0.9861$; AUDA: ${\bar{R}}^{2}=0.9956$.

Attention was focused on the *n*-butanol fractions of DCA and DCC and on the mannitol fraction obtained during the precipitation process. The samples were initially tested at concentrations of 100 and 50 μg/mL. The *n*-BuOH fractions showed higher inhibitory activity, with inhibition values of approximately 80% at 100 μg/mL and 60% at 50 μg/mL, whereas the mannitol fraction displayed lower inhibition percentages (52.0 ± 0.8% at 100 μg/mL and 43.3 ± 4.7% at 50 μg/mL) (Table 5). Subsequently, IC_50_ values were determined for all samples. Consistent with the preliminary screening results, DCA and DCC *n*-BuOH exhibited comparable COX-2 inhibitory activity, with IC_50_ values of 28.51 ± 0.36 μg/mL and 29.22 ± 0.72 μg/mL, respectively (Figure 3). In contrast, the mannitol fraction was significantly less active, showing an IC_50_ value of 74.82 ± 7.99 μg/mL (Figure 3). To assess the activity of the samples against additional enzymes involved in the arachidonic acid cascade, their inhibitory effects on soluble epoxide hydrolase (sEH) were also evaluated. In the sEH assay, all samples evidenced measurable activity (Table 5); however, DCA *n*-BuOH extract demonstrated notably inhibitory activity, with an IC_50_ value of 9.08 ± 0.28 μg/mL, followed by the mannitol fraction (IC_50_ = 13.84 ± 0.91 μg/mL) (Figure 4). Contrary to expectations, DCC *n*-BuOH extract showed lower activity, with an IC_50_ value approximately threefold higher than that of DCA *n*-BuOH extract (Figure 4). It is worth noting that the IC_50_ value of the crude extract is expressed in μg/mL due to its complex multi-component nature, and thus serves as a preliminary indicator of bioactivity rather than a micromolar concentration comparable to pure reference compounds (Figure 3 and Figure 4). A plausible explanation for this different activity may lie in the distinct metabolite composition of the two extracts. The molecular networking analysis indicated that the DCA extract contained a higher abundance and structural diversity of oxylipins and terpenoid-related metabolites than DCC.

### 2.6. In Silico Investigation of n-BuOH Fractions Against COX-2 and sEH

**Figure 5:**
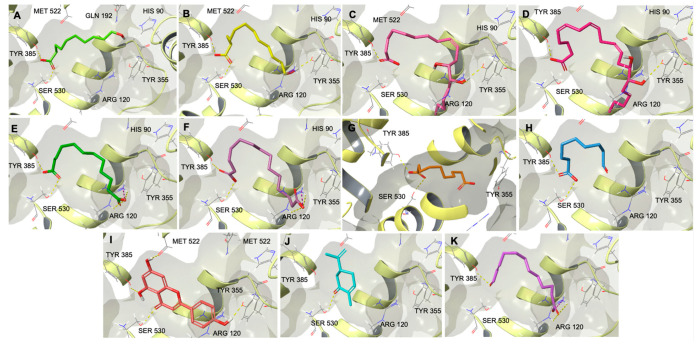
Three-dimensional binding mode of (**A**) 10-hydroxydecanoic acid (colored by atom type: C light green, O red, polar H white), (**B**) 12-hydroxydodecanoic acid (colored by atom type: C yellow, O red, polar H white), (**C**) 12,13-DHOME (colored by atom type: C pink, O red, polar H white), (**D**) 14,15-DiHETrE (colored by atom type: C magenta, O red, polar H white), (**E**) dodecanedioic acid (colored by atom type: C green, O red), (**F**) tetradecanedioic acid (colored by atom type: C mauve, O red), (**G**) Sebacic acid (colored by atom type: C orange, O red), (**H**) 9-Oxo-nonanoic acid (colored by atom type: C azure, O red), (**I**) apigenin (colored by atom type: C dark orange, O red, polar H white), (**J**) carvone (colored by atom type: C cyan, O red, polar H white), and (**K**) traumatin (colored by atom type: C purple, O red, polar H white) in the COX-2 (PDB ID: 5IKQ) [42] catalytic site. Hydrogen bonds are represented as dotted yellow lines while π–cation interactions are represented as dotted purple lines.

To rationalize, at the molecular level, the enzymatic bioactivity previously observed for the carrot seed fractions, molecular docking studies were performed by predicting the putative binding modes of individual compounds identified by LC-MS in DCA *n*-BuOH, DCC *n*-BuOH, and mannitol against two inflammation-related targets, namely COX-2 and sEH. Given the high chemical complexity of the extracts and the likelihood that the inhibitory activity observed in the cell-free assays arises from the combined contribution of multiple constituents rather than from a single dominant ligand, we focused on molecules from the major chemical classes detected, for which no previous binding mode or mechanistic rationale had been reported for either target, including fatty acid derivatives, oxylipins, flavonoids, phenolic compounds, stilbenes, terpenes, and acylcarnitines (Supplementary Table S1). For these selected compounds, LC-MS peak areas were used as a semi-quantitative estimate of their relative abundance in the extracts, in order to better understand the docking results and their possible contribution to the observed activity. As reported in Table S2, the metabolite profiles of DCA and DCC *n*-BuOH extracts were largely comparable, with the notable exception of the oxylipins 12,13-DHOME and 14,15-DiHETrE, which exhibited higher relative signal intensities in the DCA extract compared to the DCC extract. These differences may contribute to the higher inhibitory activity observed for the DCA extract. However, the biological activity of complex plant extracts cannot necessarily be attributed to individual metabolites, as additive or synergistic interactions among multiple constituents may substantially contribute to the overall biological effect [40,41]. Therefore, the higher abundance of these oxylipins should be regarded as identifying candidate metabolites associated with the observed activity rather than as direct evidence of a causal role. Regarding COX-2, the binding modes illustrated in Figure 5 and Figure S18 (Table S7) showed that the active site could accommodate several metabolites capable of establishing key interactions within the canonical catalytic pocket, particularly in proximity to Tyr385, Ser530, Arg120, His90, and Met522, similar to the known inhibitor celecoxib (Figure S18G).

Among the aliphatic derivatives, 10-hydroxydecanoic acid, 12-hydroxydodecanoic acid, 12,13-DHOME, 14,15-DiHETrE, dodecanedioic acid, tetradecanedioic acid, sebacic acid, 9-oxo-nonanoic acid, traumatin (Figure 5A–H,K) adopted an extended conformation along the cyclooxygenase channel, with their polar termini positioned to form hydrogen-bonding interactions with Ser530, Tyr385, Tyr355, and Arg120. A similar binding pattern was observed for the aromatic metabolites, especially apigenin and carvone (Figure 5I,J). These ligands displayed favorable docking poses, with the planar aromatic scaffold inserted into the hydrophobic channel and the phenolic hydroxyl and/or carbonyl groups oriented toward Tyr385 and Ser530, residues central to COX-2 catalysis and frequently engaged by known inhibitors [43].

**Figure 6:**
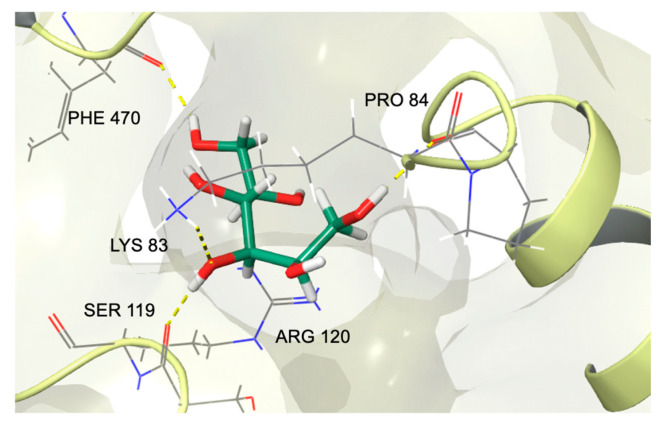
Three-dimensional binding mode of mannitol (colored by atom type: C teal, O red, polar H white) in the COX-2 (PDB ID: 5IKQ) [42] catalytic site. Hydrogen bonds are represented as dotted yellow lines.

Moreover, considering the mannitol fraction, the mannitol occupied the active-site cavity through a hydrogen-bonding network involving Phe470, Ser119, Lys83, and Pro84; however, the absence of interactions with Tyr385 and Ser530 was consistent with the lower inhibitory activity observed for this fraction (Figure 6).

Taken together, these results suggested that the stronger COX-2 inhibition displayed by the *n*-BuOH fractions could be attributed to the cooperative presence of several metabolites, structurally well-suited to engage the enzyme catalytic site.

**Figure 7:**
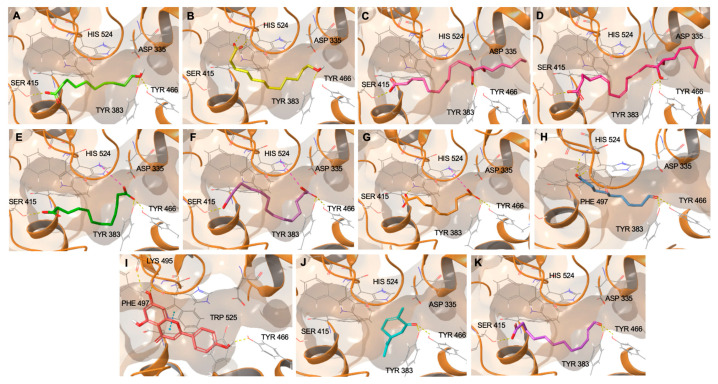
Three-dimensional binding mode of (**A**) 10-hydroxydecanoic acid (colored by atom type: C light green, O red, polar H white), (**B**) 12-hydroxydodecanoic acid (colored by atom type: C yellow, O red, polar H white), (**C**) 12,13-DHOME (colored by atom type: C pink, O red, polar H white), (**D**) 14,15-DiHETrE (colored by atom type: C magenta, O red, polar H white), (**E**) Dodecanedioic acid (colored by atom type: C green, O red), (**F**) tetradecanedioic acid (colored by atom type: C mauve, O red), (**G**) Sebacic acid (colored by atom type: C orange, O red), (**H**) 9-oxo-nonanoic acid (colored by atom type: C azure, O red), (**I**) apigenin (colored by atom type: C dark orange, O red, polar H white), (**J**) carvone (colored by atom type: C cyan, O red, polar H white), and (**K**) traumatin (colored by atom type: C purple, O red, polar H white) in the sEH (PDB ID: 3WKE) [44] catalytic site. Hydrogen bonds, π–π and π–cation interactions are represented as dotted yellow lines, blue lines and purple lines, respectively.

A similar result was observed for sEH, whose active site accommodates structurally diverse ligands through an L-shaped lipophilic pocket lined by residues that support both hydrophobic contacts and polar anchoring (Figure 7 and Figure S19; Table S8). Notably, 10-hydroxydecanoic acid, 12-hydroxydodecanoic acid, 12,13-DHOME, 14,15-DiHETrE, dodecanedioic acid, tetradecanedioic acid, sebacic acid, and 9-oxo-nonanoic acid occupied the catalytic site, with their terminal carboxylate or hydroxyl groups positioned to establish H-bond interactions with Tyr383, Tyr466, and Asp335, which constitute the catalytic triad of the enzyme (Figure 7A–H). These binding poses resemble the recognition pattern expected for endogenous substrate and are consistent with competitive occupation of the enzyme channel. Likewise, apigenin displayed favorable binding modes, reflecting the ability of its planar aromatic systems to establish hydrophobic interactions with His524 and Trp525, while its hydroxyl substituents contributed to hydrogen bonds with Tyr383 and Tyr466 (Figure 7I). Similarly, carvone and traumatin occupied the hydrophobic tunnel and formed contacts with residues involved in substrate recognition, thereby potentially restricting access of the endogenous substrate to the catalytic site (Figure 7J,K).

**Figure 8:**
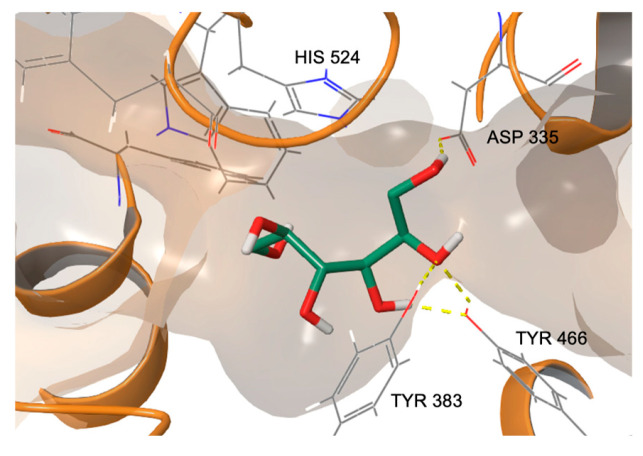
Three-dimensional binding mode of mannitol (colored by atom type: C teal, O red, polar H white) in the sEH (PDB ID: 3WKE) [44] catalytic site. Hydrogen bonds are represented as dotted yellow lines.

Regarding the mannitol fraction, as expected, mannitol showed a productive binding mode in the enzyme active site, establishing H-bonds with Tyr383, Tyr466, and Asp335 (Figure 8), consistent with the canonical binding pattern of sEH inhibitors, similar to the known inhibitor AUDA (Figure S19G) [44].

Overall, these findings support a multi-component mechanism for sEH inhibition, in which both oxygenated fatty acids and aromatic metabolites contribute to target engagement.

Taken together, these data indicate that the anti-inflammatory activity of the *n*-BuOH extract of the *D. carota* L. seed was the result of an additive and synergistic action of several constituents with complementary binding properties, supporting the potential of the *n*-BuOH fractions as sources of natural inhibitors targeting different pathways of the arachidonic acid cascade.

## 3. Materials and Methods

### 3.1. Standards and Reagents

The following reagents were purchased from Sigma-Aldrich (Milan, Italy): 1,1-diphenyl-2-picrylhydrazyl (DPPH^•^), 2,2′-azinobis(3-ethylbenzothiazoline-6-sulfonic acid) diammonium salt (ABTS), potassium persulfate, Folin–Ciocalteu reagent, gallic acid, sodium carbonate, 6-hydroxy-2,5,7,8-tetramethyltetrahydrochromane-2-carboxylic acid (Trolox), and (+)-Catechin hydrate. Analytical-grade solvents employed for the extraction and Kupchan protocols, namely methanol, chloroform, *n*-butanol, and *n*-hexane, were purchased from VWR Intl. (West Chester, PA, USA). Acetonitrile, water, and methanol of LC-MS grade (Ultra LC ROMIL Pure Chemistry, Cambridge, UK) were used for qualitative LC-MS analysis. Deuterium oxide (99.95% D, Sigma-Aldrich, Milan, Italy) was used for NMR analysis.

### 3.2. Plant Material and Extracts Preparation

Wild specimens of *Daucus carota* L. subsp. *carota* were collected in the field at two sites in the Central Apennines (Italy): Capracotta in the Molise region (DCC) and Amatrice in the Lazio region (DCA), in October 2021… Both study sites belong to the Temperate macrobioclimate with a Submediterranean variant according to the Worldwide Bioclimatic Classification System of Rivas-Martínez [45], and the soils are classified as Eutric Cambisols according to the World Reference Base for Soil Resources (WRB) [46]. After harvesting, the umbels were air-dried in the shade, then the fruits were separated and placed in closed containers. The material was identified by taxonomist Professor Paola Fortini. The voucher specimens (Amatrice IS05357; Capracotta IS05358) were deposited in the Herbarium of the University of Molise (IS).

For both samples (DCC and DCA), 68 g of dry carrot seeds were ground in a mortar and macerated in 375 mL of methanol for 2 h. The methanol was filtered and removed by rotary evaporator under reduced pressure and low temperature. The seeds were both further extracted in 250 mL of methanol twice for 4 and 24 h, respectively. The methanol extracts obtained were filtered and combined with the first methanol extract before the desiccation step. Crude extract (DCC = 4.05 g; DCA = 3.73 g) was obtained with a yield for DCC and DCA of 5.96% and 5.49%, respectively. The crude methanol extracts were then subjected to a modified Kupchan partitioning method to obtain four fractions: *n*-hexane extract, chloroform extract, *n*-butanol extract, and water extract. For comparison, an ultrasound-assisted hydroalcoholic extract (80% MeOH) was prepared, as described by Dehghani et al. with some modifications [47]. Briefly, 0.75 g of dry and ground seeds were weighed, and 12 mL of 80% methanol was added. Ultrasonication was performed for 45 min at 40 °C and a frequency of 50–60 Hz. The mixture was kept in the dark for 15 min and centrifuged at 4000 rpm for 15 min. The supernatant was recovered and evaporated to dryness using a rotary evaporator under reduced pressure and low temperature. Following the Kupchan separation to obtain the *n*-BuOH extract, we observed the formation of insoluble agglomerates during the drying process with a rotary evaporator. As these precipitates were soluble in water, they were manually collected from the *n*-BuOH extract, then washed with the same solvent and subjected to ^1^H and ^13^C NMR analysis in D_2_O. Mannitol was identified (See Supplementary Figure S9), and the NMR data were compared with the current literature and with the Human Metabolome Database (HMDB). NMR experiments were acquired on a Bruker DRX-600 spectrometer (Bruker BioSpin GmbH, Rheinstetten, Germany) at 600 MHz.

### 3.3. Total Phenolic Content (TPC)

The total phenolic content (TPC) of the hydroalcoholic (80% MeOH), water, and *n*-BuOH soluble fractions was analysed using the Folin–Ciocalteu reagent, as described by Penu et al., with some modifications [48]. Briefly, the dry extracts were dissolved in methanol, and 500 µL of the solution was added to a test tube containing 500 µL of Folin–Ciocalteu reagent, previously diluted ten-fold with deionized water. The mixture was left to stand for 5 min. Subsequently, 3 mL of 7% sodium carbonate and 1 mL of water were added. The test solution was incubated for 1 h to allow color development and then centrifuged for 3 min. Absorbance was read at 765 nm using a UV-Vis spectrophotometer (Shimadzu UV-1601 spectrophotometer, Kyoto,Japan). Deionized water was used as a blank, while gallic acid was used as the external standard for the calibration curve (R^2^ = 0.999). Results were expressed as gallic acid equivalents (GAE) per gram of dry extract (mg GAE/g DE). All measurements were performed in triplicate.

### 3.4. Total Flavonoid Content (TFC)

The total flavonoid content (TFC) was analysed with a spectrophotometric assay, as described by Akhtar et al. with modifications [49]. In particular, an aliquot (200–1000 µL) of the hydroalcoholic (80% MeOH), water, and *n*-BuOH soluble fractions was dissolved in methanol and diluted with an adequate volume of deionised water. Then, 80 µL of 5% sodium nitrite solution was added, and the mixture was left to stand for 6 min. After this period, 150 µL of aluminum chloride was added. After 6 min in the dark, 500 µL of 1 M sodium hydroxide was added. Absorbance was measured at 510 nm using a UV-Vis spectrophotometer (Shimadzu UV-1601 spectrophotometer, Kyoto, Japan). Deionized water was used as a blank, while catechin was used as the external standard for the calibration curve (R^2^ = 0.999). Results were expressed as catechin equivalents (CE) per gram of dry extract (mg CE/g DE). All analyses were performed in triplicate.

### 3.5. Antioxidant Capacity

The antioxidant capacity of the hydroalcoholic extract (80% MeOH) and the fractions obtained by modified Kupchan partitioning was evaluated using two complementary spectrophotometric assays: the DPPH radical scavenging and the ABTS radical cation decolorization assays.

#### 3.5.1. DPPH Radical Scavenging Assay

DPPH radical scavenging activity was determined according to Dehghani et al. (2025) [47], with minor modifications. A DPPH working solution was prepared by dissolving 2 mg of DPPH in 50 mL of methanol, corresponding to a concentration of 0.04 mg/mL (approximately 0.10 mM). Before use, the solution was diluted with methanol to obtain an absorbance of approximately 0.78 at 517 nm. An aliquot of 50 µL of each appropriately diluted sample was mixed with 1.0 mL of the DPPH working solution. The mixture was incubated in the dark for 30 min at room temperature, and absorbance was measured at 517 nm (Shimadzu UV-1601 spectrophotometer, Shimadzu Corporation, Kyoto, Japan). A reagent control was prepared by replacing the sample with 50 µL of methanol. Trolox was used as the external standard for the calibration curve (R^2^ = 0.999). Results were expressed as milligrams of Trolox equivalents (TE) per gram of dry extract (mg TE/g DE). All measurements were performed in triplicate.

#### 3.5.2. ABTS Radical Cation Scavenging Assay

ABTS radical scavenging activity was determined according to [50], with minor modifications. The ABTS radical cation (ABTS^•+^) stock solution was generated by mixing an aqueous ABTS solution (7 mM) with potassium persulfate (2.45 mM). The mixture was incubated in the dark at room temperature for 12–16 h to allow radical formation. Before analysis, the ABTS^•+^ stock solution was diluted with methanol to obtain an absorbance of 0.70 at 734 nm. An aliquot of 15 µL of each appropriately diluted sample was added to 150 µL of methanol and 1.35 mL of ABTS^•+^ working solution. A reagent control was prepared by replacing the sample with methanol, while methanol was used as the blank. The reaction mixture was incubated in the dark for 30 min at room temperature, and absorbance was then measured at 734 nm (Shimadzu UV-1601 spectrophotometer, Shimadzu Corporation, Kyoto, Japan). Trolox was used as the external standard for the calibration curve (R^2^ = 0.999). Results were expressed as milligrams of Trolox equivalents (TE) per gram of dry extract (mg TE/g DE). All measurements were performed in triplicate.

### 3.6. Elemental Composition Analysis

Microwave-assisted acid digestion was performed for the elemental analysis of *D. carota* seeds, as described by Menezes et al. with some modifications [51]. Briefly, 0.3 g of dry seeds was weighed into PTFE vessels, to which 4 mL of concentrated nitric acid (HNO_3_), 2 mL of hydrogen peroxide (H_2_O_2_), and 4 mL of ultrapure water (H_2_O) were added. Vessels were placed in the microwave system (ETHOS EASY, Milestone, Sorisole, Italy) and subjected to the following program: 5 min at 1800 W to reach 125 °C, 5 min at a constant temperature of 125 °C, 10 min at 1800 W to increase the temperature from 125 °C to 180 °C, and 5 min at a constant temperature of 180 °C. After cooling, the solutions were filtered using a PTFE syringe filter (0.45 µm), transferred to a volumetric flask, and diluted to a final volume of 25 mL with ultrapure water. Samples were analysed using an inductively coupled plasma optical emission spectrometer (ICP-OES) (5800 ICP-OES, Agilent, Santa Clara, CA, USA). Quantification was performed using the external calibration method. In particular, standard solutions of 100 mg L^−1^ of iron (Fe), potassium (K), phosphorus (P), and sulphur (S), and a standard solution of 10 mg L^−1^ of copper (Cu), magnesium (Mg), manganese (Mn), boron (B), barium (Ba), nickel (Ni), and zinc (Zn), both dissolved in 10% nitric acid (HNO_3_) and supplied by Supelco (Multielement standard solution 5 for ICP and Metalloid and non-metal mix for ICP), were used.

Data are expressed as mean ± standard deviation of three independent biological replicates (*n* = 3).

### 3.7. High-Resolution Mass Spectrometric Analysis and Molecular Mapping of Daucus Phytochemicals

An HPLC system (Ultimate 3000, Thermo Fisher Scientific, Bremen, Germany) connected to an Orbitrap Q-Exactive Classic mass spectrometer (Thermo Fisher Scientific, Bremen, Germany) was used to analyze each extract. LC-MS analysis was conducted following the procedure described by Fantasma et al. (2026) [52], with minor modifications. A Luna Omega C18 column (3 µm, 150 × 2.1 mm) (Phenomenex, Torrance, CA, USA) was used for metabolite separation. Phase A was H_2_O with 0.1% formic acid, and Phase B was CH_3_CN with 0.1% formic acid. A gradient program was run from 5% to 95% of Phase B over 30 min, with a 5 µL full-loop injection. The system included an electrospray ionization source that switched between positive and negative ion modes. Each extract was subjected to full-ion MS and fragmentation using a data-dependent scan. The following were the operating parameters for both positive and negative ion modes: sheath and auxiliary gas flow (N_2_), 40 and 5; sweep gas, 0; spray voltage, 5; capillary temperature, 275 °C; FTMS scan mode with a mass range of 150 to 1500 *m*/*z* and a resolution of 70,000.

Compound Discoverer software version 3.3 was used for the initial interpretation and identification of metabolites. The hydroalcoholic extract, a broad representation of polar and semi-polar constituents of *D. carota* L., was subjected to metabolite mapping using the molecular networking node in the Compound Discoverer™ v. 2.2 (Thermo Fisher Scientific, Bremen Germany) workflow. Data processing was performed using the ‘Untargeted Food Research ID with Statistics’ workflow, which integrates RT alignment, background subtraction, and multi-level identification (mzCloud, ChemSpider, and mzLogic), followed by differential statistical analysis to pinpoint potential markers of the two accessions. Peak areas of the metabolites selected for molecular docking were obtained from the LC-MS profiles using Xcalibur software (version 2.2) and are reported in Table S2.

### 3.8. Cell-Free COX-2 Activity Assay

The inhibitory effects of the extracts on COX-2 activity were assessed using the COX-2 Inhibitor Screening Kit (Fluorometric, ab283401), as already reported by us [52,53]. Each extract was tested at a final concentration of 100 and 50 μg/mL in triplicate under single-concentration conditions. For the assay, 8 μL of the extract solutions, celecoxib (used as a control), or vehicle solution (buffer containing 2% DMSO) were added to a reaction mixture consisting of 2 μL of cofactor solution (1:200 dilution), 1 μL of COX probe, 76 μL of assay buffer, and 1 μL of COX-2 enzyme. The mixture was incubated for 10 min at 25 °C prior to substrate addition. Enzyme activity was then initiated by introducing 10 μL of arachidonic acid substrate, previously activated with NaOH and diluted tenfold before use. The resulting fluorescence signal was recorded as Relative Fluorescence Units (RFU) at excitation and emission wavelengths of 535 nm and 587 nm, respectively, using an EnSpire™ Multimode Plate Reader (PerkinElmer, Santa Clarita, CA, USA). For DCA *n*-BuOH extract, DCC *n*-BuOH extract, and Mannitol, the IC_50_ values were calculated by testing a series of 2-fold serial dilutions spanning concentrations from 200 to 0.195 µg/mL. On the other hand, for celecoxib, ${\mathrm{IC}}_{50}$ value was determined using a series of 3-fold serial dilutions spanning concentrations from $30\times 10^{-6}$ to $5\times 10^{-10}\;M$ (or 30 µM to 0.5 nM).

### 3.9. Cell-Free sEH Activity Assay

Using the Soluble Epoxide Hydrolase Inhibitor Screening Assay Kit (Cayman: 10011671) and in accordance with the protocol previously implemented in our laboratory [52,54], the chosen fractions were first assessed for their inhibitory effect on sEH at fixed concentrations of 100 and 50 µg/mL in triplicate. IC_50_ values were calculated by testing a series of 2-fold serial dilutions spanning concentrations from 200 to 0.195 µg/mL. For AUDA, ${\mathrm{IC}}_{50}$ value was determined using a series of 3-fold serial dilutions spanning concentrations from $10\times 10^{-6}$ to $1\times 10^{-11}\;M$ (or 10 µM to 0.01 nM).

Briefly, recombinant human sEH was diluted in 25 mM bis-Tris buffer (pH 7.0) and incubated for 15 min at 25 °C in the presence of either the investigated extracts, the standard inhibitor AUDA, or vehicle control (2.5% DMSO). The enzymatic reaction was started by adding 50 µL of the diluted substrate (401039), then the fluorescence intensity was recorded after 60 min at excitation and emission wavelengths of 330 nm and 465 nm, using an EnSpire™ Multimode Plate Reader (PerkinElmer, Foster City, CA, USA).

### 3.10. Molecular Docking Experiments

The three-dimensional structures of the target proteins, namely cyclooxygenase-2 (PDB ID: 5IKQ) [42] and soluble epoxide hydrolase (PDB ID: 3WKE) [44], co-crystallized with known inhibitors, were retrieved from the Protein Data Bank (PDB) and prepared using the Protein Preparation Workflow [55,56] implemented in the Schrödinger Suite: all solvent molecules and co-crystallized ligands were removed, cap termini and missing hydrogen atoms were introduced, bond orders were assigned, and protonation states were optimized at physiological pH.

Receptor grids for molecular docking were generated by centering the grid on the centroid of the co-crystallized ligand to define the active site region.

Regarding COX-2, the grid box center was set at coordinates x = 21.60, y = 51.88, z = 17.69, with inner and outer box dimensions of 20 × 20 × 20 Å and 50 × 50 × 50 Å, respectively. For sEH, the grid box center was defined at coordinates x = −16.77, y = −8.13, z = 66.27, with inner and outer box dimensions of 10 × 10 × 10 Å and 28.51 × 28.51 × 28.51 Å, respectively.

The 2D structures of the main chemical constituents were drawn using the 2D Sketcher (Schrödinger Suite 2025-2) and subsequently prepared with LigPrep [57], which generated all possible tautomers and protonation states at pH 7.4 ± 1.0, followed by energy minimization.

Molecular docking experiments were performed with Glide software version 10.7 [58,59,60,61,62] (Schrödinger Suite 2025-2) in the Extra Precision (XP) mode. During the initial sampling phase, 10,000 poses were generated for each ligand. Subsequently, 800 conformations were retained for energy minimization, and the top 30 poses were selected for detailed analysis of protein-ligand interactions and identification of the most favorable binding modes within the enzyme active sites.

### 3.11. Statistical Analysis

Data are reported as mean ± standard deviation (SD). For TPC, TFC, DPPH, and ABTS, the effects of geographical origin/population and extraction solvent were evaluated using two-way analysis of variance (ANOVA), with geographical origin/population, solvent fraction, and their interaction included as fixed factors. For DPPH and ABTS, the analysis included the five tested preparations: *n*-hexane, chloroform, *n*-butanol, 80% methanol, and the aqueous fraction. For TPC and TFC, the analysis was restricted to the *n*-butanol, 80% methanol, and aqueous samples because measurements in the n-hexane and chloroform fractions could not be reliably determined due to lipid interference. When significant effects were detected, multiple comparisons among means were performed using Tukey’s honestly significant difference test.

Differences between DCC and DCA in DPPH and ABTS activity were additionally assessed within each solvent fraction using two-tailed unpaired Student’s *t*-tests. To account for the five pairwise comparisons conducted for each assay, *p*-values were adjusted separately using the Holm method. Differences were considered statistically significant when the Holm-adjusted *p*-value was below 0.05.

For elemental composition, differences between DCA and DCC were assessed separately for each element using a two-tailed unpaired Student’s *t*-test. To account for multiple testing across the 11 elements analysed, the resulting *p*-values were adjusted using the Benjamini–Hochberg false discovery rate procedure. Adjusted *p*-values below 0.05 were considered statistically significant.

## 4. Conclusions

The present study provided a comprehensive qualitative characterization of the phytochemical composition and potential biological properties of seed extracts and fractions obtained from two wild *Daucus carota* L. subsp. *carota* accessions collected in two different Italian geographical areas. The combined metabolomic, biological, and in silico approaches highlighted a clear relationship between chemical composition and bioactivity, demonstrating that the extraction solvent strongly influenced the distribution of bioactive metabolites and, consequently, the antioxidant and anti-inflammatory potential of the obtained fractions.

Qualitative LC-MS-based metabolomic profiling revealed a largely conserved core metabolome between the two accessions, dominated by oxylipins, polar phospholipid derivatives, flavonoids, and terpenoid-related metabolites. Nevertheless, significant differences were observed in the abundance and diversity of oxylipins and terpene-derived compounds, which were particularly enriched in the Amatrice accession. The *n*-BuOH fractions were characterized by the highest levels of total phenolics and flavonoids and exhibited the strongest antioxidant activity, confirming that compounds responsible for radical scavenging preferentially partition into solvents of intermediate polarity. The anti-inflammatory assays further demonstrated the biological relevance of the *n*-BuOH fractions, which selectively inhibited key enzymes involved in the arachidonic acid cascade, including COX-2 and sEH.

Although the two accessions displayed distinct inhibition profiles toward these targets, both fractions showed promising activity, supporting their potential as natural modulators of inflammatory pathways. Molecular docking analyses corroborated these findings, indicating that multiple metabolites identified in the active fractions may contribute to the observed effects through complementary interactions with the target enzymes.

The anti-inflammatory activity may reflect the combined effects of the identified compounds; however, further studies are needed to clarify whether additive or synergistic interactions occur. The integration of metabolomic profiling, enzymatic assays, and molecular docking provided new insights into the compounds potentially responsible for these activities and supports the valorization of wild carrot seeds as promising ingredients for the development of nutraceutical, cosmeceutical, and health-promoting products. Further studies aimed at isolating the most active constituents and validating their efficacy in cellular and in vivo models will be necessary to fully elucidate their mechanisms of action and potential applications.

Overall, this study presents the first phytochemical and biological characterization of *Daucus carota* subsp. carota seeds accessions collected from a native population in the Molise region, thereby expanding current knowledge of the chemical diversity and biological potential of this wild matrix. More broadly, this work contributes to the scientific documentation and valorization of the largely unexplored floristic heritage of Molise, one of the most biodiversity-rich Italian regions. The present study also highlights the importance of integrating biodiversity conservation with phytochemical and pharmacological research, thereby promoting the responsible exploration and preservation of wild plant resources in the context of ongoing environmental change.

## Figures and Tables

**Table 1:** Total phenolic content (TPC) and total flavonoid content (TFC) of the 80% MeOH extract and the *n*-hexane, chloroform, *n*-BuOH, and aqueous fractions obtained by modified Kupchan partitioning of *Daucus carota* seed from Capracotta (DCC) and Amatrice (DCA). TPC is expressed as milligrams of gallic acid equivalents per gram of dry extract (mg GAE/g DE), whereas TFC is expressed as milligrams of catechin equivalents per gram of dry extract (mg CE/g DE).

Fraction	DCC		DCA	
	TPC (mg GAE/g DE)	TFC (mg CE/g DE)	TPC (mg GAE/g DE)	TFC (mg CE/g DE)
*n*-Hexane	n.d.	n.d.	n.d.	n.d.
Chloroform	n.d.	n.d.	n.d.	n.d.
*n*-BuOH	38.49 ± 1.31 ^a^	38.32 ± 2.80 ^a^	32.18 ± 1.57 ^b^	28.07 ± 0.65 ^b^
80% MeOH	15.59 ± 0.41 ^d^	19.12 ± 1.03 ^c^	19.45 ± 1.22 ^c^	36.27 ± 0.95 ^a^
Water	5.10 ± 0.25 ^e^	1.04 ± 0.09 ^d^	7.10 ± 0.36 ^e^	1.82 ± 0.13 ^d^

Values are expressed as mean ± standard deviation (*n* = 3). Within each response variable, means followed by different superscript letters are significantly different according to Tukey’s honestly significant difference test (*p* < 0.05). Statistical comparisons for TPC and TFC were restricted to the *n*-BuOH, 80% MeOH, and aqueous fraction because measurements in the *n*-hexane and chloroform fractions could not be reliably determined due to lipid interference. n.d., not determined; DE, dry extract.

**Table 2:** Antioxidant activity of the 80% MeOH extract and the *n*-hexane, chloroform, *n*-BuOH, and aqueous fractions obtained by modified Kupchan partitioning of *Daucus carota* L. seed from Capracotta (DCC) and Amatrice (DCA), evaluated by DPPH and ABTS assays. Results are expressed as milligrams of Trolox equivalents per gram of dry extract (mg TE/g DE).

Fraction	DCC		DCA	
	DPPH (mg TE/g DE)	ABTS (mg TE/g DE)	DPPH (mg TE/g DE)	ABTS (mg TE/g DE)
*n*-Hexane	0.28 ± 0.06 ^g^	1.00 ± 0.44 ^f^	0.38 ± 0.02 ^g^	1.55 ± 0.40 ^f^
Chloroform *	9.93 ± 0.25 ^e^	23.28 ± 1.80 ^e^	11.50 ± 0.47 ^e^	22.82 ± 1.09 ^e^
*n*-BuOH ***	88.04 ± 1.50 ^a^	118.02 ± 9.88 ^a^	64.00 ± 0.82 ^b^	80.08 ± 0.59 ^b^
80% MeOH *	14.78 ± 0.60 ^d^	36.40 ± 4.41 ^d^	17.08 ± 0.13 ^c^	47.14 ± 3.73 ^cd^
Water	4.20 ± 0.28 ^f^	48.59 ± 3.12 ^c^	4.22 ± 0.42 ^f^	40.93 ± 2.24 ^cd^

Values are expressed as mean ± standard deviation (*n* = 3). Within each antioxidant assay, means followed by different superscript letters are significantly different according to Tukey’s honestly significant difference test (*p* < 0.05). Means sharing at least one superscript letter are not significantly different. DE, dry extract. Asterisks indicate statistically significant differences between DCC and DCA within the same solvent fraction for the DPPH assay, as determined by a two-tailed Student’s *t*-test followed by Holm correction for multiple comparisons: * *p* < 0.05 and *** *p* < 0.001. No significant differences were observed for the ABTS assay after Holm correction.

**Table 3:** Mineral composition of *Daucus carota* seeds from Capracotta (DCC) and Amatrice (DCA), expressed as mg kg^−1^ dry matter. Values are expressed as mean ± SD of three independent biological replicates (*n* = 3). For each element, differences between DCA and DCC were assessed using a two-tailed unpaired Student’s *t*-test. To account for multiple testing across the 11 elements analysed, the resulting *p*-values were adjusted using the Benjamini–Hochberg procedure. * adjusted *p* < 0.05, ** adjusted *p* < 0.01, and *** adjusted *p* < 0.001.

Element	DCA	DCC
	Mean ± SD (mg kg^−1^ DM)	
B *** (λ 249.772 nm)	24.02 ± 0.85	15.41 ± 0.92
Ba *** (λ 455.403 nm)	52.39 ± 1.04	30.21 ± 0.55
Cu (λ 327.395 nm)	12.00 ± 0.79	14.1 ± 0.98
Fe *** (λ 259.940 nm)	123.09 ± 6.54	242.46 ± 16.84
K ** (λ 766.491 nm)	7573.33 ± 468.77	4853.89 ± 105.67
Mg * (λ 280.270 nm)	2894.46 ± 193.18	2406.19 ± 42.92
Mn * (λ 257.610 nm)	54.35 ± 3.26	69.32 ± 6.13
Ni *** (λ 216.555 nm)	3.23 ± 0.23	1.41 ± 0.13
P ** (λ 213.618 nm)	6498.01 ± 372.77	4747.58 ± 81.76
S (λ 181.972 nm)	2338.38 ± 102.13	2178.00 ± 26.67
Zn (λ 206.200 nm)	51.67 ± 3.63	47.22 ± 0.48

**Table 4:** Metabolites identified in hydroalcoholic extracts (80% methanol) of carrot seeds from the Amatrice (DCA) and Capracotta (DCC) accessions by LC-MS analysis in positive and negative ionization modes. Metabolite annotation was performed using Compound Discoverer software version 2.2.

Name	Formula	ppm	m/z	RT [min]	Reference Ion	MS/MS
10-Hydroxy-2-decenoic acid	C_10_H_18_O_3_	−3.43	185.118	15.813	[M−H]^−1^	59.0127/141.0910/167.0705
12.13-DHOME	C_18_H_34_O_4_	0.56	295.2280	26.857	[M-H−H_2_O]^−1^	277.2170/251.2379/155.1433
13-HODE	C_18_H_32_O_3_	−0.36	295.2280	28.378	[M−H]^−1^	277.2172/251.2378/193.1591/151.0832
16-Hydroxyhexadecanoic acid	C_16_H_32_O_3_	0.45	271.2280	29.453	[M−H]^−1^	225.2220/165.4310
3-Hydroxytetradecanedioic acid	C_14_H_26_O_5_	0.77	273.1710	16.291	[M−H]^−1^	213.1491/169.0860/87.0075
5-Phenylvaleric acid	C_11_H_14_O_2_	0.06	179.1070	21.754	[M+H]^+1^	151.0752/119.0492/97.9916/56.9654
Adenosine	C_10_H_13_N_5_O_4_	0.41	268.1040	2.596	[M+H]^+1^	136.0616
Arginine	C_6_H_14_N_4_O_2_	0.68	175.1190	1.663	[M+H]^+1^	116.0707/130.0974/158.0923/70.0657
Azelaic acid	C_9_H_16_O_4_	−3.5	187.0970	13.21	[M−H]^−1^	125.0960/169.0860
Caryophyllene oxide	C_15_H_24_O	0.2	221.1900	29.602	[M+H]^+1^	203.1794/175.1481/161.1323
chlorogenic acid	C_16_H_18_O_9_	1.3	353.0880	9.2	[M−H]^−1^	191.0555
Citric acid	C_6_H_8_O_7_	−3.59	191.0190	1.891	[M−H]^−1^	111.0077/129.0183/87.0076/173.0084
Dodecanedioic acid	C_12_H_22_O_4_	−1.13	229.1440	16.414	[M−H]^−1^	183.1383/211.1340
Elemicin	C_12_H_16_O_3_	3.18	207.1022	17.94	[M−H]^−1^	161.0450/73.0282.218.9920/192.0419
Ethyl oleate	C_20_H_38_O_2_	−1.35	311.2940	30.261	[M+H]^+1^	161.0958/135.0440/177.0543/109.1013
Mannitol	C_6_H_14_O_6_	−4.17	181.0710	1.78	[M−H]^−1^	101.0233/163.0604/89.0232/71.0126/59.0127
Galactonic acid	C_6_H_12_O_7_	−3.98	195.0500	1.772	[M−H]^−1^	177.0397/059.0290/129.0183/75.0075
Hexadeca-4.7.10.13-tetraenoic acid	C_16_H_24_O_2_	−0.09	249.1850	27.802	[M+H]^+1^	161.1323/119.0856/105.0701/81.0703
Hexadecanedioate	C_16_H_28_O_4_	0.61	283.1920	21.249	[M−H]^−1^	239.2014/265.1809/221.0907
Hexadecanedioic Acid	C_16_H_30_O_4_	0.56	285.2070	23.89	[M−H]^−1^	267.0/1965/223.2063
Indoleacrylic acid	C_11_H_9_NO_2_	0.48	188.0710	7.578	[M+H]^+1^	170.0598/146.0599/118.0692
Isoferulic acid	C_10_H_10_O_4_	−3.16	193.0500	14.901	[M−H]^−1^	149.0599/123.0437/107.0490
Isoflavone derivative	C_20_H_18_O_4_	−0.05	323.1280	22.514	[M+H]^+1^	291.1016/231.0804/161.0596/137.0597
Isoimperatorin	C_16_H_14_O_4_	0.27	271.0970	22.929	[M+H]^+1^	151.0388/147.0439/225.0906/253.0849
Linoleic acid	C_18_H_32_O_2_	0.22	279.2330	25.889	[M−H]^−1^	112.9839/96.9589
Luteolin	C_15_H_10_O_6_	0.06	285.0400	15.726	[M−H]^−1^	135.0076/049.0598/217.0511
Luteolin glucoside	C_21_H_20_O_11_	1.3	447.0935	12.13	[M−H]^−1^	285.0405
Luteolin rutinoside	C_27_H_30_O_15_	1.5	593.1516	11.67	[M−H]^−1^	285.0405
LysoPC(0:0/18:2)	C_26_H_50_NO_7_P	−0.53	520.3390	28.367	[M+H]^+1^	184.0430/104.1073/337.2737
Methyl linolenate	C_19_H_32_O_2_	−0.61	293.2470	33.226	[M+H]^+1^	205.1560/149.0231
sn-1-Monolinoleoylglycerol (18:2/0:0/0:0)	C_21_H_38_O_4_	0.71	355.2840	26.012	[M+H]^+1^	109.1013/151.1480/123.1168
Myristicin	C_11_H_12_O_3_	0.56	193.0860	11.161	[M+H]^+1^	165.0909/128.0193/134.0439
Neocnidilide	C_12_H_18_O_2_	0.62	195.1380	16.408	[M+H]^+1^	137.0961/121.1012/177.1273
Oleic acid	C_18_H_34_O_2_	−0.19	281.2490	33.139	[M−H]^−1^	161.4496
Palmitic acid	C_16_H_32_O_2_	−0.29	255.2330	32.237	[M−H]^−1^	219.8456/143.0703
Phaseolic acid	C_12_H_22_O_6_	0.53	261.1350	12.449	[M−H]^−1^	187.0970/125.0961
Phellopterin	C_17_H_16_O_5_	0.12	301.1070	23.677	[M+H]^+1^	233.0946/96.9684/78.9578
Phenylalanine	C_9_H_11_NO_2_	1.04	166.0860	4.277	[M+H]^+1^	120.0808/84.9601/103.0546
Phytosphingosine	C_18_H_39_NO_3_	0.18	318.3000	19.823	[M+H]^+1^	256.2633/113.0599/
Pinocembrin	C_15_H_12_O_4_	−0.43	255.0660	18.485	[M−H]^−1^	211.1700/153.0184/135.0078/119.0471
Sesquiterpene derivative	C_15_H_20_O	−0.02	217.1590	21.09	[M+H]^+1^	157.1011/199.1481/145.011
Sphinganine	C_18_H_39_NO_2_	0.41	302.3050	22.209	[M+H]^+1^	201.1638/137.0961/106.0865
Suberic acid	C_8_H_14_O_4_	−4.25	173.0810	11.249	[M−H]^−1^	111.0804/129.0910/61.9872
Tetradecanedioic acid	C_14_H_26_O_4_	0.16	257.1760	21.193	[M−H]^−1^	239.1649/195.1749/153.0911
Trihydroxyoctadecadienoic acid	C_18_H_32_O_5_	1.07	327.2180	16.404	[M−H]^−1^	309.2073/265.2172/197.1173/165.0556
Tryptophan	C_11_H_12_N_2_O_2_	1.04	205.0970	7.577	[M+H]^+1^	188.0705/146.0599/
Tyrosine methylester	C_10_H_13_NO_3_	−3.52	194.0820	22.441	[M−H]^−1^	151.0392/133.3593
Umbelliferone	C_9_H_6_O_3_	1.04	163.0390	18.431	[M+H]^+1^	107.0857/81.0703

**Table 5:** Inhibition percentage of DCA *n*-BuOH extract, DCC *n*-BuOH extract, and Mannitol at different concentrations against the isolated COX-2 and sEH enzymes. Celecoxib and AUDA are reported as control inhibitors. Data are expressed as means ± SD of three independent experiments repeated on three different days using the same extract stock solution, with each concentration measured in technical triplicate. Because no independent extract preparations were available, these repetitions should be regarded as technical/inter-assay replicates rather than biological replicates.

	COX-2 Inhibition Percentage ± SD		sEH Inhibition Percentage ± SD	
Samples	[Compound] 100 µg/mL	[Compound] 50 µg/mL	[Compound] 100 µg/mL	[Compound] 50 µg/mL
DCA *n*-BuOH extract	81.4 ± 0.13	60.3 ± 0.25	89.3 ± 0.1	88.9 ± 0.1
DCC *n*-BuOH extract	77.0 ± 0.33	64.5 ± 0.41	81.4 ± 0.0	71.5 ± 6.7
Mannitol	52.0 ± 0.8	43.3 ± 4.7	78.8 ± 0.3	71.2 ± 1.0
Celecoxib (COX-2 known inhibitor)	≥100	≥100	\	\
AUDA (sEH known inhibitor)	\	\	≥100	≥100

## Data Availability

The original contributions presented in this study are included in the article/supplementary material. Further inquiries can be directed to the corresponding authors.

## Supplementary Materials

The following supporting information can be downloaded at: https://www.mdpi.com/article/10.3390/plants15152400/s1, Figure S1. LC-MS profiles of 80% methanol extract from DCA (A,B) and DCC (C,D) in positive (A,C) and negative (B,D) ion modes; Figure S2. LC-MS profile of DCA (*Daucus carota* L., Amatrice) *n*-hexane extract in positive (A) and negative (B) ion mode; Figure S3: LC-MS profile of DCC (*Daucus carota* L., Capracotta) *n*-hexane extract in positive (A) and negative (B) ion mode; Figure S4: LC-MS profile of DCA (*Daucus carota* L., Amatrice) chloroform extract in positive (A) and negative (B) ion mode; Figure S5: LC-MS profile of DCC (*Daucus carota* L., Capracotta) chloroform extract in positive (A) and negative (B) ion mode; Figure S6: LC-MS profile of DCA (*Daucus carota* L., Amatrice *n*-butanol extract in positive (A) and negative (B) ion mode; Figure S7: LC-MS profile of DCC (*Daucus carota* L., Capracotta) *n*-butanol extract in positive (A) and negative (B) ion mode; Figure S8: LC-MS profile of DCA (*Daucus carota* L., Amatrice) water extract in positive (A) and negative (B) ion mode; Figure S9: LC-MS profile of DCC (*Daucus carota* L., Capracotta) water extract in positive (A) and negative (B) ion mode; Table S1: Metabolites identified in *n*-butanolic extracts of carrot seeds from the Amatrice (DCA) and Capracotta (DCC) accessions by LC-ESI-MS/MS analysis in positive and negative ionization modes. Metabolite annotation was performed using Compound Discoverer software; Table S2: Relative signal intensity of selected metabolites identified in *n*-butanolic extracts of carrot seeds from the Amatrice (DCA) and Capracotta (DCC) accessions; Table S3: Metabolites identified in water extracts of carrot seeds from the Amatrice (DCA) and Capracotta (DCC) accessions by LC-ESI-MS/MS analysis in positive and negative ionization modes. Metabolite annotation was performed using Compound Discoverer software; Table S4: Metabolites identified in *n*-hexane extracts of carrot seeds from the Amatrice (DCA) and Capracotta (DCC) accessions by LC-ESI-MS/MS analysis in positive and negative ionization modes. Metabolite annotation was performed using Compound Discoverer software; Table S5: Metabolites identified in chloroform extracts of carrot seeds from the Amatrice (DCA) and Capracotta (DCC) accessions by LC-ESI-MS/MS analysis in positive and negative ionization modes. Metabolite annotation was performed using Compound Discoverer software; Below (S10–S15) are reported ^1^H and 13C NMR spectra (1D and 2D) of mannitol (600 MHz, D_2_O); Figure S10: ^1^H NMR spectrum of mannitol (600 MHz, D_2_O); Figure S11: Enlarged region of ^1^H NMR spectrum of mannitol (600 MHz, D_2_O); Figure S12: TOCSY NMR spectrum of mannitol (400 MHz, D_2_O); Figure S13: Enlarged region of HSCQ NMR spectrum of mannitol (600 MHz, D_2_O); Figure S14: Overlap of an enlarged region of HMBC (green) and HSQC (blu and red) NMR spectra of mannitol (600 MHz, D2O); Figure S15: DEPT-135° NMR spectrum of mannitol (400 MHz, D_2_O); Table S6: ^1^H and ^13^C NMR assignments of Mannitol (D2O, 600 MHz); Figure S16: Chemical structure of mannitol; Figure S17: Visual chemical shifts assignment of mannitol referenced to δ_H_ TMS = 0. Figure S18: 3D binding mode of (A) dihydrokaempferol (colored by atom type: C light purple, O red, polar H white), (B) ferulic acid 4-O-sulfate (colored by atom type: C blue, O red, S yellow), (C) acetovanillone (colored by atom type: C violet, O red, polar H white), (D) rhapontigenin (colored by atom type: C dark violet, O red, polar H white), (E) isovalerylcarnitine (colored by atom type: C lime, O red, N blue), (F) O-pimeloylcarnitine (colored by atom type: C lilac, O red, N blue) and (G) celecoxib (colored by atom type: C pink, O red, N blue) in the COX-2 (PDB ID: 5IKQ) catalytic site. Hydrogen bonds and π–cation interactions are represented as dotted yellow and dotted green lines; Table S7: Docking score reported by the selected compounds against COX-2; Figure S19: 3D binding mode of (A) ferulic acid 4-*O*-sulfate (colored by atom type: C blue, O red, S yellow), (B) acetovanillone (colored by atom type: C violet, O red, polar H white), (C) rhapontigenin (colored by atom type: C dark violet, O red, polar H white), (D) isovalerylcarnitine (colored by atom type: C lime, O red, N blue), (E) O-pimeloylcarnitine (colored by atom type: C lilac, O red, N blue), (F) dihydrokaempferol (colored by atom type: C light purple, O red, polar H white), and (G) AUDA (colored by atom type: C salmon, O red, N blue) in the sEH (PDB ID: 3WKE) catalytic site. Hydrogen bonds, π–π and π–cation interactions are represented as dotted yellow lines, blue lines and purple lines, respectively; Table S8. Docking score reported by the selected compounds against sEH.
