# Supplementary material for: Exploring the Anti-Inflammatory Potential of Daucus carota L. subsp. carota Seed Extracts: Phytochemical Profiling, In Vitro Antioxidant Activity and Modulation of the Arachidonic Acid Cascade
Source: Plants (Basel). 2026 Aug 5;15(15):2400. doi: 10.3390/plants15152400 (PMC13468354; doi:10.3390/plants15152400)
Supplement: Supplementary file 1 [file plants-15-02400-s001.zip › plants-4442261-supplementary.pdf]

Supporting Information

Exploring the Anti-Inflammatory Potential of *Daucus carota* L. subsp. *carota* Seed Extracts: Phytochemical Profiling, *In Vitro* Antioxidant Activity and Modulation of the Arachidonic Acid Cascade

Monica Maio[1,±], Gilda D'Urso[2,±], Alessandra Capuano[2], Francesca Fantasma[1], Michela Aliberti[2], Ester Colarusso[2], Gabriella Saviano[1], Vincenzo De Felice[1], Paola Fortini[1], Gianluigi Lauro[2], Maria Giovanna Chini[1,*], Agostino Casapullo[2], Giuseppe Bifulco[2,*,†] and Maria Iorizzi[1,†]

[1] Department of Biosciences and Territory, University of Molise, Contrada Fonte Lappone, 86090 Isernia, Italy; m.maio3@studenti.unimol.it (M.M.); fantasma@unimol.it (F.F.); saviano@unimol.it (G.S.); defelice@unimol.it (V.D.F.); fortini@unimol.it (P.F.); iorizzi@unimol.it (M.I.)

[2] Department of Pharmacy, University of Salerno, Via Giovanni Paolo II 132, 84084 Salerno, Italy; gidurso@unisa.it (G.D.); acapuano@unisa.it (Al.C.), mialiberti@unisa.it (M.A.); ecolarusso@unisa.it (E.C.); glauro@unisa.it (G.L.); casapullo@unisa.it (A.C.).

[±]These authors contributed equally. [†]Co-last authorship

*Correspondence: ; mariagiovanna.chini@unimol.it (M.G.C.); bifulco@unisa.it (G.B.)

Tel.: +39-0874404132 (M.G.C.); +39-089969741 (G.B.);

Table of contents

Figure S1. LC-MS profiles of 80% methanol extract from DCA (A, B) and DCC (C, D) in positive (A, C) and negative (B, D) ion modes. 4

Figure S2. LC-MS profile of DCA (*Daucus carota* L., Amatrice) *n*-hexane extract in positive (A) and negative (B) ion mode....... 5

Figure S3. LC-MS profile of DCC (*Daucus carota* L., Capracotta) *n*-hexane extract in positive (A) and negative (B) ion mode....... 6

Figure S4. LC-MS profile of DCA (*Daucus carota* L., Amatrice) chloroform extract in positive (A) and negative (B) ion mode....... 7

Figure S5. LC-MS profile of DCC (*Daucus carota* L., Capracotta) chloroform extract in positive (A) and negative (B) ion mode....... 8

Figure S6. LC-MS profile of DCA (*Daucus carota* L., Amatrice) *n*-butanol extract in positive (A) and negative (B) ion mode....... 9

Figure S7. LC-MS profile of DCC (*Daucus carota* L., Capracotta) *n*-butanol extract in positive (A) and negative (B) ion mode....... 10

Figure S8. LC-MS profile of DCA (*Daucus carota* L., Amatrice) water extract in positive (A) and negative (B) ion mode 11

Figure S9. LC-MS profile of DCC (*Daucus carota* L., Capracotta) water extract in positive (A) and negative (B) ion mode....... 12

Table S1: Metabolites identified in *n*-butanolic extracts of carrot seeds from the Amatrice (DCA) and Capracotta (DCC) accessions by LC-ESI-MS/MS analysis in positive and negative ionization modes. Metabolite annotation was performed using Compound Discoverer software. 13

Table S2: Relative signal intensity of selected metabolites identified in *n*-butanolic extracts of carrot seeds from the Amatrice (DCA) and Capracotta (DCC) accessions 16

Table S3: Metabolites identified in water extracts of carrot seeds from the Amatrice (DCA) and Capracotta (DCC) accessions by LC-ESI-MS/MS analysis in positive and negative ionization modes. Metabolite annotation was performed using Compound Discoverer software 17

Table S4: Metabolites identified in *n*-hexane extracts of carrot seeds from the Amatrice (DCA) and Capracotta (DCC) accessions by LC-ESI-MS/MS analysis in positive and negative ionization modes. Metabolite annotation was performed using Compound Discoverer software 18

Table S5: Metabolites identified in chloroform extracts of carrot seeds from the Amatrice (DCA) and Capracotta (DCC) accessions by LC-ESI-MS/MS analysis in positive and negative ionization modes. Metabolite annotation was performed using Compound Discoverer software 19

Below **(S10 -S15)** are reported ^{1}H and ^{13}C NMR spectra (1D and 2D) of mannitol (600MHz, D_2O). 20

Figure S10: ^{1}H NMR spectrum of mannitol (600MHz, D_2O). 20

Figure S11: Enlarged region of ^{1}H NMR spectrum of mannitol (600MHz, D_2O). 20

Figure S12: TOCSY NMR spectrum of mannitol (400MHz, D_2O). 21

Figure S13: Enlarged region of HSCQ NMR spectrum of mannitol (600MHz, D_2O). 21

Figure S14: Overlap of an enlarged region of HMBC (green) and HSQC (blu and red) NMR spectra of mannitol (600MHz, D_2O). 22

Figure S15: DEPT-135° NMR spectrum of mannitol (400MHz, D_2O). 22

Table S6 ^{1}H and ^{13}C NMR assignments of Mannitol (D_2O, 600 MHz) 23

Figure S16: Chemical structure of mannitol. 23

Figure S17: Visual chemical shifts assignment of mannitol referenced to δ_H TMS = 0 23

Figure S18. 3D binding mode of A) dihydrokaempferol (colored by atom type: C light purple, O red, polar H white), B) ferulic acid 4-O-sulfate (colored by atom type: C blue, O red, S yellow), C) acetovanillone (colored by atom type: C violet, O red, polar H white), D) rhapontigenin (colored by atom type: C dark violet, O red, polar H white), E) isovalerylcarnitine (colored by atom type: C lime, O red, N blue), F) O-pimeloylcarnitine (colored by atom type: C lilac, O red, N blue) in the COX-2 (PDB ID: 5IKQ) catalytic site. Hydrogen bonds are represented as dotted yellow lines. .. 24

Table S7. Docking score reported by the selected compounds against COX-2. 24

Figure S19. 3D binding mode of A) ferulic acid 4-O-sulfate (colored by atom type: C blue, O red, S yellow), B) acetovanillone (colored by atom type: C violet, O red, polar H white), C) rhapontigenin (colored by atom type: C dark violet, O red, polar H white), D) isovalerylcarnitine (colored by atom type: C lime, O red, N blue), E) O-

pimeloylcarnitine (colored by atom type: C lilac, O red, N blue) in the sEH (PDB ID: 3WKE) catalytic site. Hydrogen bonds, π- π and π-cation interactions are represented as dotted yellow lines, blue lines and purple lines, respectively. 26

Table S8. Docking score reported by the selected compounds against sEH..26

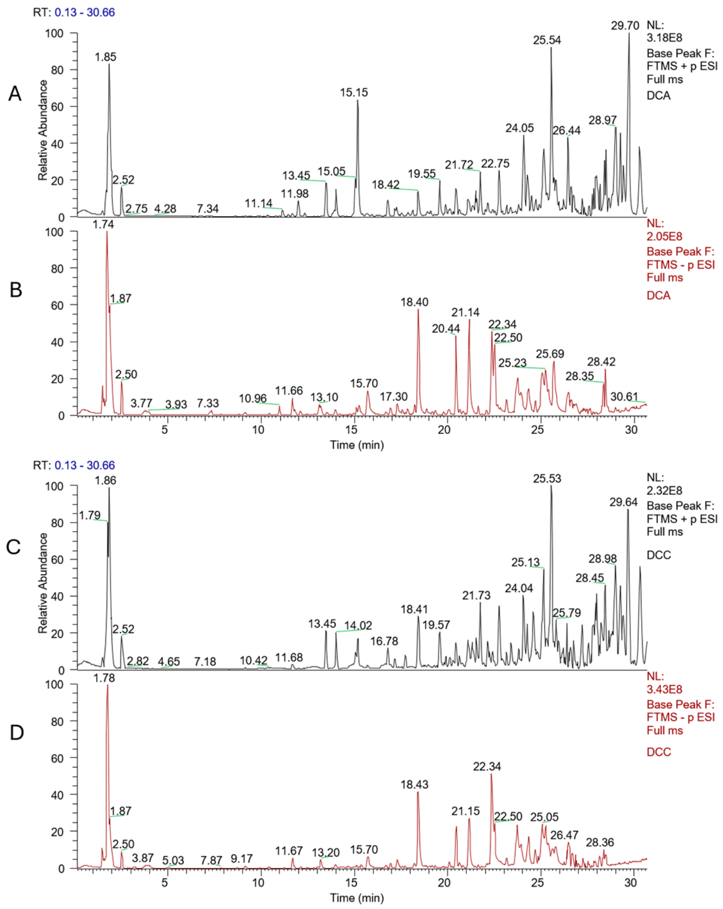

Figure S1. LC-MS profiles of 80% methanol extract from DCA (A, B) and DCC (C, D) in positive (A, C) and negative (B, D) ion modes.

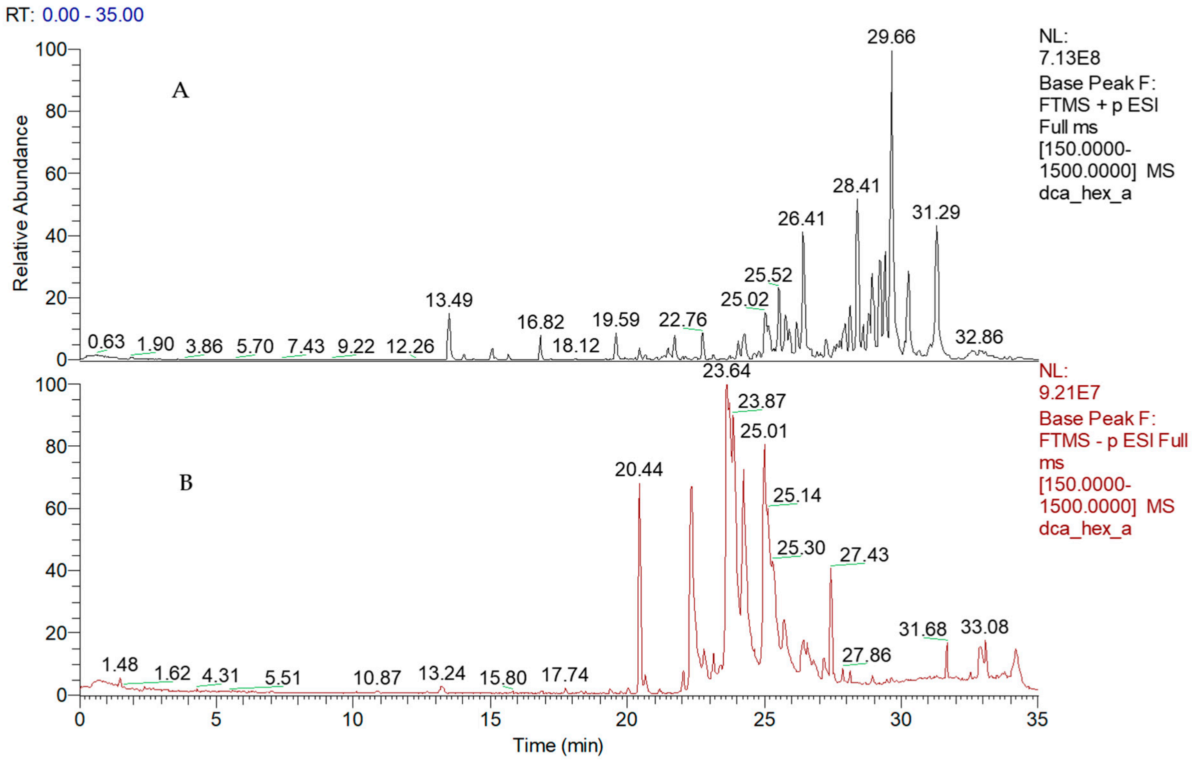

Figure S2. LC-MS profile of DCA (*Daucus carota* L., Amatrice) *n*-hexane extract in positive (A) and negative (B) ion mode

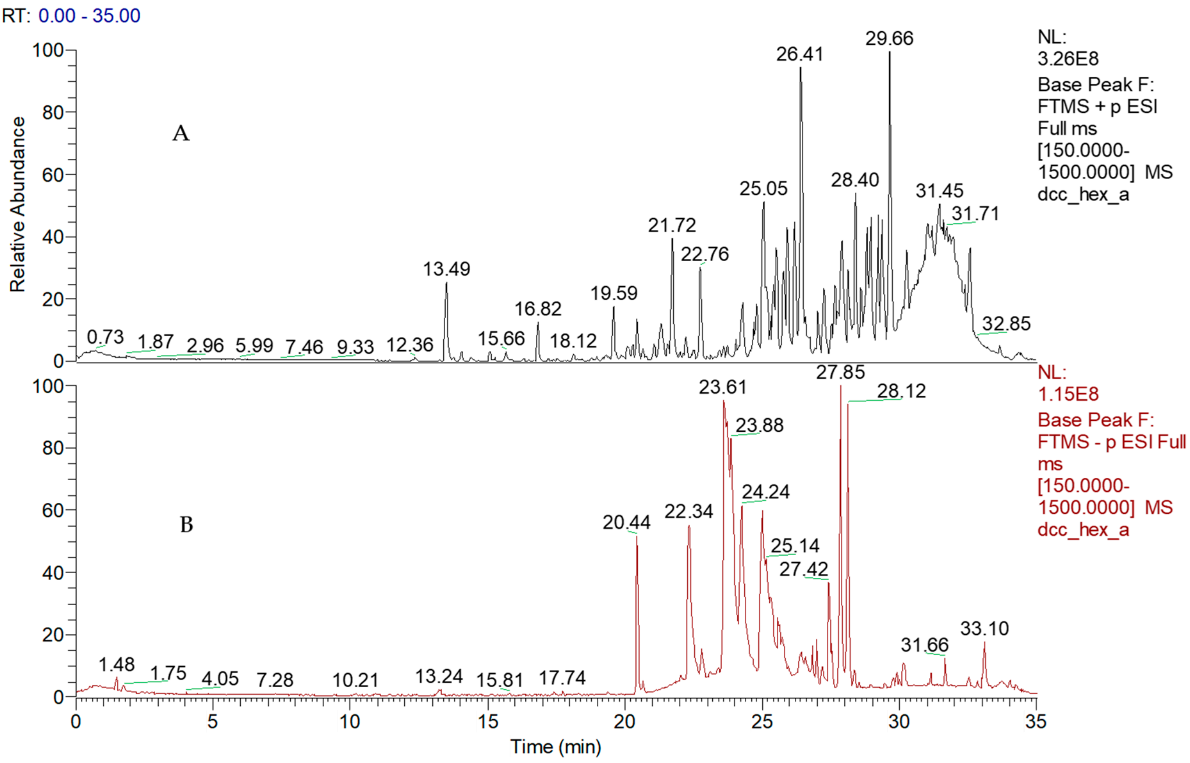

Figure S3. LC-MS profile of DCC (*Daucus carota* L., Capracotta) *n*-hexane extract in positive (A) and negative (B) ion mode

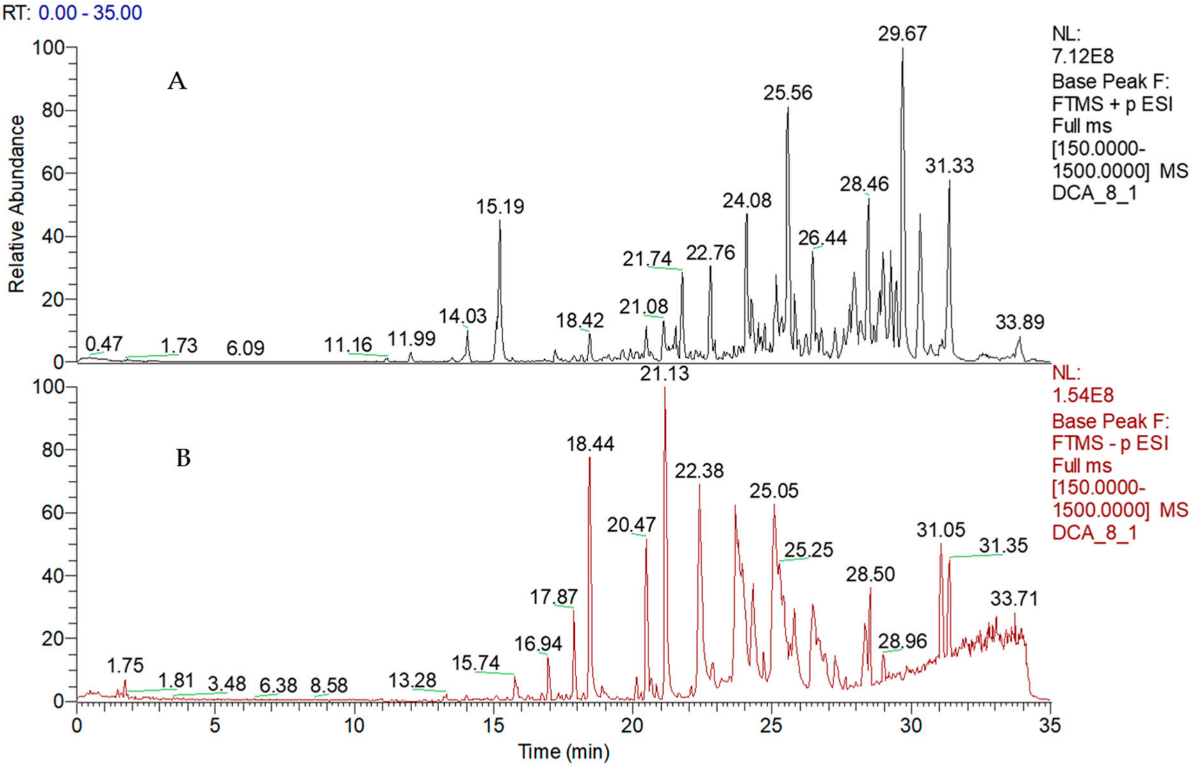

Figure S4. LC-MS profile of DCA (*Daucus carota* L., Amatrice) chloroform extract in positive (A) and negative (B) ion mode

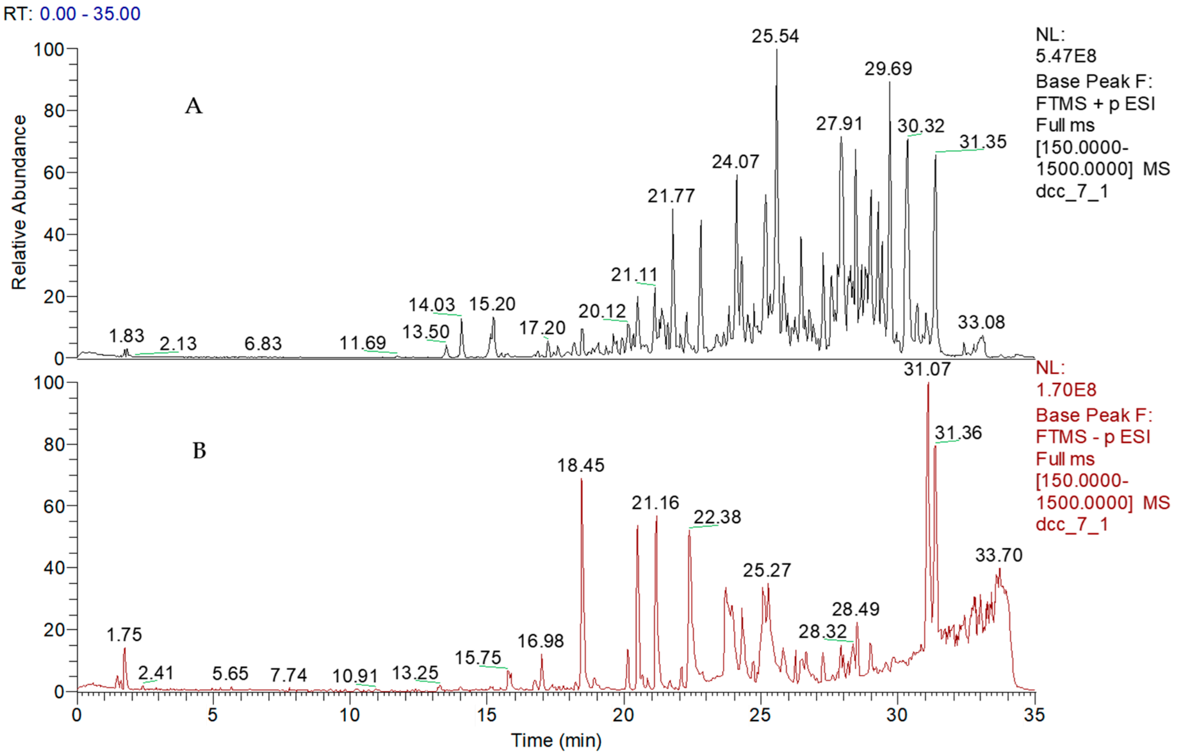

Figure S5. LC-MS profile of DCC (*Daucus carota* L., Capracotta) chloroform extract in positive (A) and negative (B) ion mode

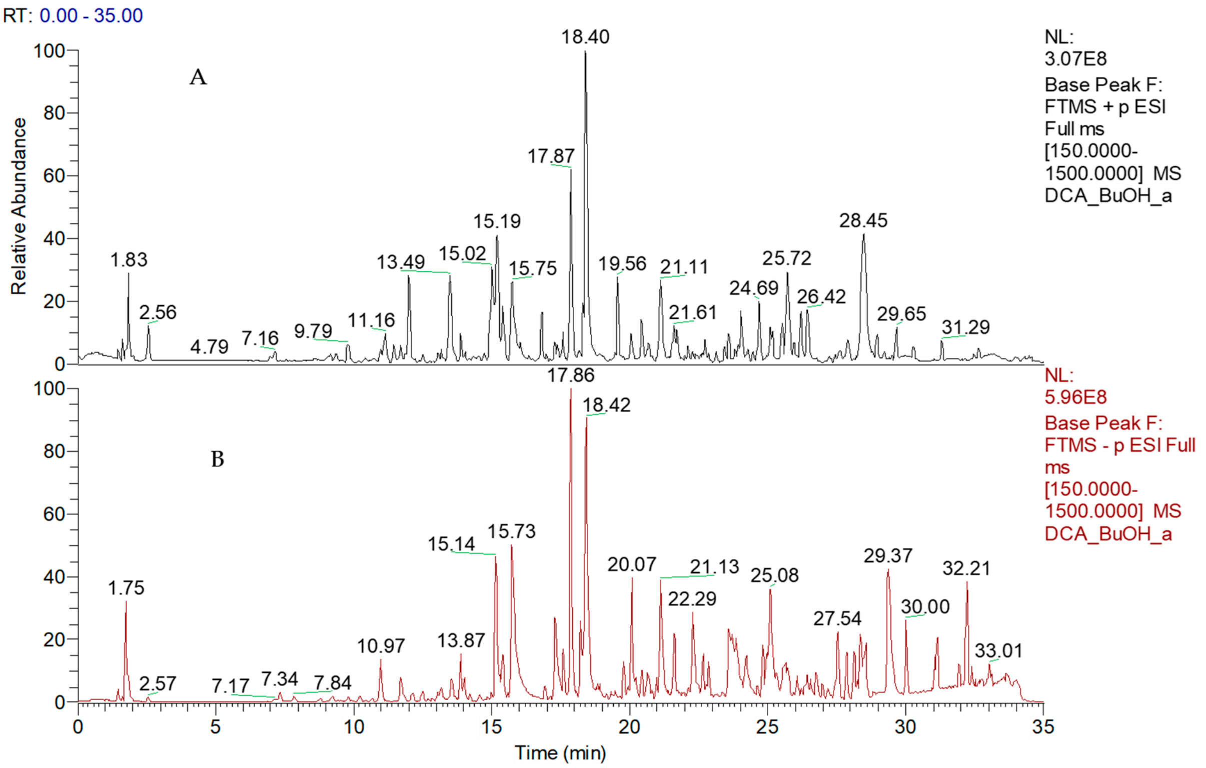

Figure S6. LC-MS profile of DCA (*Daucus carota* L., Amatrice) *n*-butanol extract in positive (A) and negative (B) ion mode

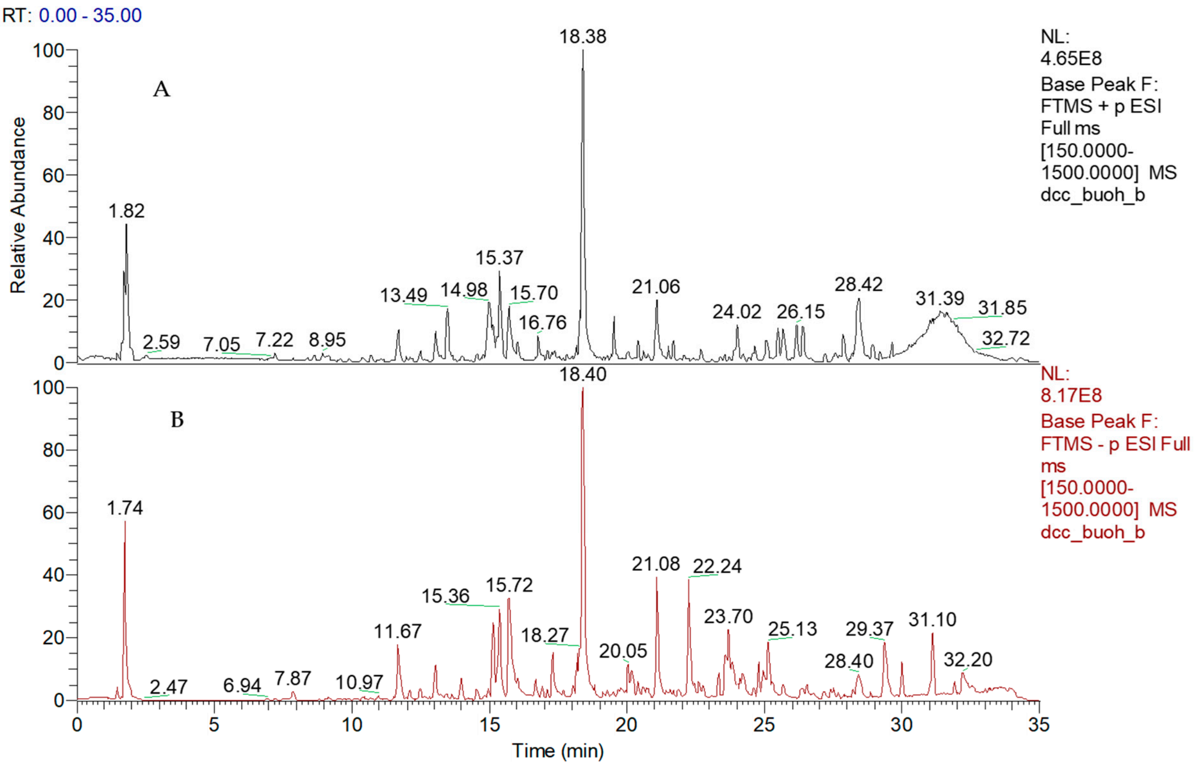

Figure S7. LC-MS profile of DCC (*Daucus carota* L., Capracotta) *n*-butanol extract in positive (A) and negative (B) ion mode

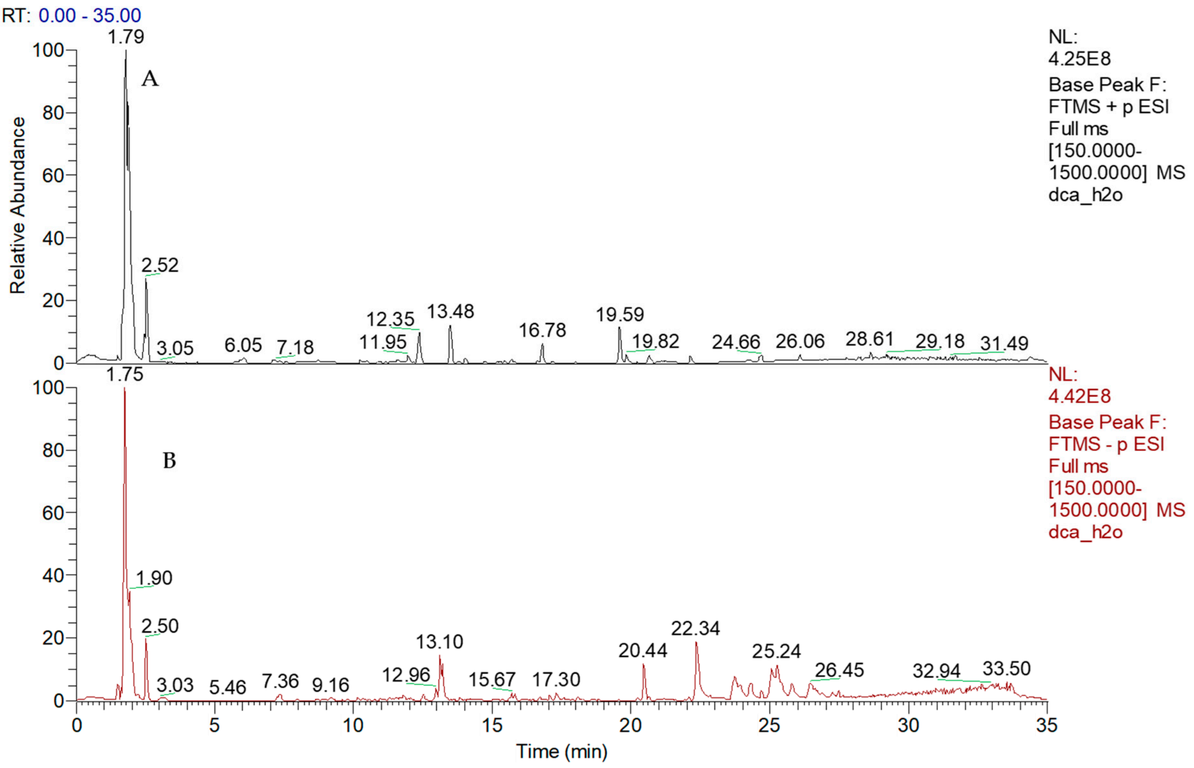

Figure S8. LC-MS profile of DCA (*Daucus carota* L., Amatrice) water extract in positive (A) and negative (B) ion mode

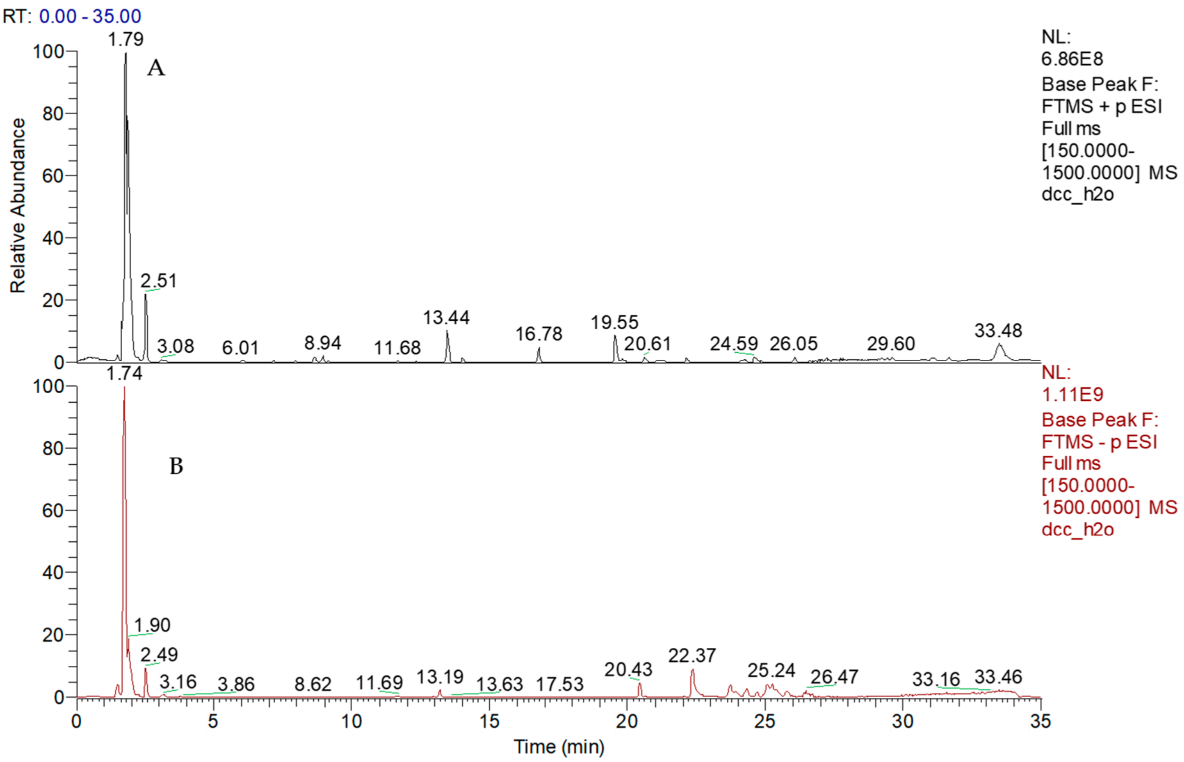

Figure S9. LC-MS profile of DCC (*Daucus carota* L., Capracotta) water extract in positive (A) and negative (B) ion mode

Table S1: Metabolites identified in *n*-butanolic extracts of carrot seeds from the Amatrice (DCA) and Capracotta (DCC) accessions by LC-ESI-MS/MS analysis in positive and negative ionization modes. Metabolite annotation was performed using Compound Discoverer software.

Name	Formula	ppm	m/z	RT [min]	Reference Ion	MS/MS
(2-Acetyloxy-3-hydroxypropyl) -octadec-9-enoate	$C_{23}H_{42}O_5$	-1.39	397.296	33.096	$[M-H]^{-1}$	183.011
10-Hydroxy-2-decenoic acid	$C_{10}H_{18}O_3$	-3.92	185.118	15.821	$[M-H]^{-1}$	59.0126
10-Hydroxydecanoic acid	$C_{10}H_{20}O_3$	-2.91	187.133	19.786	$[M-H]^{-1}$	141.1275/125.0961
12,13-DHOME	$C_{18}H_{34}O_4$	-0.43	295.228	27.855	$[M-H-H_2O]^{-1}$	197.1905/249.2222/141.1275
12-Hydroxydodecanoic acid	$C_{12}H_{24}O_3$	-1.4	215.165	23.226	$[M-H]^{-1}$	169.1589
13-HODE	$C_{18}H_{32}O_3$	-0.56	295.228	28.356	$[M-H]^{-1}$	195.1383/277.2172/183.1019/171.1017
14,15-DiHETrE	$C_{20}H_{34}O_4$	0.97	337.239	21.925	$[M-H]^{-1}$	319.2278/217.0505/119.0492
16-Hydroxyhexadecanoic acid	$C_{16}H_{32}O_3$	0.46	271.228	28.236	$[M-H]^{-1}$	253.2171/225.2220/235.2066/197.1900
2-Hydroxy-6-pentadecylbenzoic acid	$C_{22}H_{36}O_3$	-0.47	349.274	23.821	$[M+H]^{+1}$	331.2625/303.2679/19.1428
2-Hydroxymyristic acid	$C_{14}H_{28}O_3$	-0.81	243.196	26.404	$[M-H]^{-1}$	197.1904
3-Hydroxycapric acid	$C_{10}H_{20}O_3$	-3.71	187.133	18.521	$[M-H]^{-1}$	141.1275/125.0961
3-Hydroxy-tetradecanoic acid	$C_{14}H_{28}O_3$	-0.9	243.196	25.202	$[M-H]^{-1}$	197.1905
4,7-Dihydroxy-2H-1-benzopyran-2-one	$C_9H_6O_4$	-2.52	177.019	9.498	$[M-H]^{-1}$	133.0285/105.0333
4-Methoxycinnamaldehyde	$C_{10}H_{10}O_2$	-1.23	163.075	26.21	$[M+H]^{+1}$	105.0336/100.0245
5,10-Pentadecadien-1-ol	$C_{15}H_{28}O$	-2.03	223.206	28.12	$[M-H]^{-1}$	177.1276/166.0627/87.0076
7-Epi-12-hydroxyjasmonic acid	$C_{12}H_{18}O_4$	-1.11	225.113	17.523	$[M-H]^{-1}$	207.1021/181.1227/163.11222/137.0962
8,11,14-Heptadecatrienal	$C_{17}H_{28}O$	-0.34	249.221	22.818	$[M+H]^{+1}$	109.1013/69.0704/137.0959
9-Hydroxylinoleic acid	$C_{18}H_{32}O_3$	-0.65	295.228	25.097	$[M-H]^{-1}$	277.2172/195.1383/171.1017
9-Oxo-nonanoic acid	$C_9H_{16}O_3$	-2.19	171.102	14.263	$[M-H]^{-1}$	141.0911/127.0754
Acetovanillone	$C_9H_{10}O_3$	-0.21	167.07	13.888	$[M+H]^{+1}$	139.0753/121.1012/84.9601
Adenosine	$C_{10}H_{13}N_5O_4$	-0.26	268.104	2.579	$[M+H]^{+1}$	136.0618
apigenin	$C_{15}H_{10}O_5$	1.05	269.046	15.13	$[M-H]^{-1}$	133.0284
Apigenin 7-sulfate	$C_{15}H_{10}O_8S$	0.67	349.003	14.607	$[M-H]^{-1}$	269.0455/135.0440
Aplotaxene	$C_{17}H_{28}$	-2.2	233.226	28.529	$[M+H]^{+1}$	121.1012/135.1167
Arginine	$C_6H_{14}N_4O_2$	0.24	175.119	1.658	$[M+H]^{+1}$	116.0707/130.0975/158.0923/70.0657/60.0562
Azelaic acid	$C_9H_{16}O_4$	-3.51	187.097	13.217	$[M-H]^{-1}$	125.0961/169.0863
Camphor	$C_{10}H_{16}O$	0.4	153.127	12.871	$[M+H]^{+1}$	135.1166/107.0856/93.0702
Carvone	$C_{10}H_{14}O$	0.66	151.112	20.298	$[M+H]^{+1}$	87.0043/98.5122/128.0191
Caryophyllene oxide	$C_{15}H_{24}O$	-0.66	203.179	29.647	$[M+H-H_2O]^{+1}$	147.1167/161.1323/119.0856/109.1014

Coniferyl aldehyde	$C_{10}H_{10}O_3$	0.12	179.07	14.006	[M+H]+1	147.0438/119.0491/
Deoxyadenosine	$C_{10}H_{13}N_5O_3$	0.05	252.109	2.885	[M+H]+1	153.0696/98.9845
Dihydrokaempferol	$C_{15}H_{12}O_6$	0.64	287.056	11.49	[M-H]-1	269.0457/225.0552/163.0029/149.0235/109.0283
Dodecanedioic acid	$C_{12}H_{22}O_4$	-1.36	229.144	17.391	[M-H]-1	169.1226/141.1274/59.0127
Ethyl oleate	$C_{20}H_{38}O_2$	-1.65	311.294	30.228	[M+H]+1	149.0964/183.0112
Farcarindiol	$C_{17}H_{25}O_2$	1.5	261,1854	25.12	[M-H]-1	105.0701/81.0703/119.0856/161.1323
fenilpropanoid derivative	$C_{17}H_{26}O_4$	0.01	277.18	20.239	[M+H-H2O]+1	137.0596/111.0442
Ferulic acid 4-O-sulfate	$C_{10}H_{10}O_7S$	0.65	273.008	11.912	[M-H]-1	135.0078/151.0392/119.0492/255.0663
Mannitol	$C_6H_{14}O_6$	-3.74	181.071	1.749	[M-H]-1	136.9088/92.9187
Glucosylsphingosine	$C_{24}H_{47}NO_7$	0.04	460.328	23.943	[M-H]-1	271.2279/442.3174/225.2222
Hexadeca-4,7,10,13-tetraenoic acid	$C_{16}H_{24}O_2$	0.24	249.185	27.562	[M+H]+1	171.1165/189.1272/107.0857
Hexadecanedioic acid	$C_{16}H_{30}O_4$	0.28	285.207	23.965	[M-H]-1	135.0076/149.0599
Indoleacrylic acid	$C_{11}H_9NO_2$	0.07	188.071	7.586	[M+H]+1	146.0599/118.0652
Isoambrettolide	$C_{16}H_{28}O_2$	-0.44	253.216	18.219	[M+H]+1	99.0443/151.0751/123.0805
Isoferulic acid	$C_{10}H_{10}O_4$	-3.13	193.05	14.9	[M-H]-1	161.0234/137.0233
isoflavon derivative	$C_{15}H_{14}O_3$	-0.54	241.087	13.423	[M-H]-1	135.0441
isoflavone derivative	$C_{15}H_{10}O_4$	0.11	253.051	22.816	[M-H]-1	191.1436/165.1276
Isoimperatorin	$C_{16}H_{14}O_4$	-0.03	271.096	22.886	[M+H]+1	203.0334/69.0704
Isotaxiresinol	$C_{19}H_{22}O_6$	0.53	345.135	13.419	[M-H]-1	330.1109/193.0499/151.0390
Isovalerylcarnitine	$C_{12}H_{23}NO_4$	-0.13	246.17	8.974	[M+H]+1	116.9274/200.8805/135.0425
Linoleic acid	$C_{18}H_{32}O_2$	-0.15	279.233	25.87	[M-H]-1	235.0528/117.1199/96.9588
Luteolin	$C_{15}H_{10}O_6$	-0.47	285.04	15.738	[M-H]-1	135.0077/149.0598/053.0184/270.0535
luteolin arabinosil glucopiranoside	$C_{26}H_{28}O_{15}$		579.1359	11.55	[M-H]-1	2.850.405
Luteolin rutinoside	$C_{27}H_{30}O_{15}$		593.1301	12.01	[M-H]-1	285.0405//447.0783/
Myristicin	$C_{11}H_{12}O_3$	0	225.112	11.155	[M+H+MeOH]+1	193.0857/210.08883/165.0907/151.0752
Neocnidilide	$C_{12}H_{18}O_2$	0.06	195.138	13.962	[M+H]+1	177.1271/137.0960/113.9637
9 nitrooctadecenoic Acid	$C_{18}H_{33}NO_4$	-0.06	328.248	19.958	[M+H]+1	176.1279/98.9844
Noreugenin	$C_{10}H_8O_4$	0.29	193.05	12.27	[M+H]+1	165.0909/105.0702/128.0193
O-Pimeloylcarnitine	$C_{14}H_{25}NO_6$	-0.46	304.175	17	[M+H]+1	135.1169/107.0858/93.0702
Palmitic acid	$C_{16}H_{32}O_2$	-0.82	255.233	32.208	[M-H]-1	167.1072/123.0803

Pentadecanal	$C_{15}H_{30}O$	-0.18	271.228	29.372	[M+FA-H]-1	225.2219/177.9403/119.00036
Phaseolic acid	$C_{12}H_{22}O_6$	0.04	261.134	12.453	[M-H]-1	187.0968/125.0960
Pinocembrin	$C_{15}H_{12}O_4$	-0.78	255.066	18.472	[M-H]-1	135.0078/119.0492/153.0184
Rhapontigenin	$C_{15}H_{14}O_4$	0.42	257.082	13.375	[M-H]-1	149.0234/185.1653/210.9490/239.0709
Sebacic acid	$C_{10}H_{18}O_4$	-3.27	201.113	9.238	[M-H]-1	141.0911/123.0803/97.0647/59.0127
Sesquiterpenoid derivative	$C_{15}H_{20}O$	-0.2	217.159	21.387	[M+H]+1	159.1166/199.1478/131.0855/
Sphinganine	$C_{18}H_{39}NO_2$	-0.3	302.305	22.194	[M+H]+1	159.1165.106.0865
Sphingosine	$C_{18}H_{37}NO_2$	-1.93	282.279	26.434	[M+H-H2O]+1	83.0859/97.1014/69.0704/247.2413
Stearidonic acid	$C_{18}H_{28}O_2$	0.04	277.216	21.802	[M+H]+1	135.1167/121.1012/1070857/93.0701
Tetradecanedioic acid	$C_{14}H_{26}O_4$	0.1	257.176	21.159	[M-H]-1	239.1650/195.1748
Traumatin	$C_{12}H_{20}O_3$	-0.79	213.148	17.327	[M+H]+1	195.0648/141.0030
Trihydroxyoctadecadienoic acid	$C_{18}H_{32}O_5$	0.86	327.218	16.499	[M-H]-1	171.1019/137.0963/211.1335/229.1442/309.2070
Tryptophan	$C_{11}H_{12}N_2O_2$	-0.16	205.097	7.577	[M+H]+1	188.0706/149.0232/146.0599
Tyrosine methylester	$C_{10}H_{13}NO_3$	-2.97	194.082	22.403	[M-H]-1	149.0959
Vanillactic acid	$C_{10}H_{12}O_5$	-1.82	211.061	7.36	[M-H]-1	181.0500/163.0393/153.0549
Vanilpyruvic acid	$C_{10}H_{10}O_5$	-2.28	209.045	10.622	[M-H]-1	165.0185/133.0283

Table S2: Relative signal intensity of selected metabolites identified in *n*-butanolic extracts of carrot seeds from the Amatrice (DCA) and Capracotta (DCC) accessions

Name	Formula	DCA	DCC
10-Hydroxydecanoic acid	$C_{10} H_{20} O_3$	2.09e7	3.79e7
12,13-DHOME	$C_{18} H_{34} O_4$	1.80e8	2.90e7
12-Hydroxydodecanoic acid	$C_{12} H_{24} O_3$	3.40e7	4.02e7
14,15-DiHETrE	$C_{20} H_{34} O_4$	5.50e7	1.25e6
9-Oxo-nonanoic acid	$C_9 H_{16} O_3$	1.40e7	1.20e7
Acetovanillone	$C_9 H_{10} O_3$	1.35e7	6.05e6
apigenin	$C_{15}H_{10}O_5$	2.16e9	1.50e9
Carvone	$C_{10} H_{14} O$	2.36e7	2.34e7
Dihydrokaempferol	$C_{15} H_{12} O_6$	9.30e6	1.50e7
Dodecanedioic acid	$C_{12} H_{22} O_4$	3.14e7	2.46e7
Ferulic acid 4-*O*-sulfate	$C_{10} H_{10} O_7 S$	2.19e7	8.22e6
Mannitol	$C_6 H_{14} O_6$	1.08e9	1.60e9
Isovalerylcarnitine	$C_{12} H_{23} N O_4$	5.90e6	9.40e6
O-Pimeloylcarnitine	$C_{14} H_{25} N O_6$	7.30e6	1.60e7
Rhapontigenin	$C_{15} H_{14} O_4$	9.50e6	2.20e7
Sebacic acid	$C_{10} H_{18} O_4$	5.13e7	1.80e7
Tetradecanedioic acid	$C_{14} H_{26} O_4$	2.20e7	2.50e7
Traumatin	$C_{12} H_{20} O_3$	1.60e7	1.48e7

Table S3: Metabolites identified in water extracts of carrot seeds from the Amatrice (DCA) and Capracotta (DCC) accessions by LC-ESI-MS/MS analysis in positive and negative ionization modes. Metabolite annotation was performed using Compound Discoverer software

Name	Formula	[ppm]Annot. DeltaMass	m/z	RT [min]	Reference Ion	MS/MS
10-Hydroxy-2-decenoic acid	$C_{10} H_{18} O_3$	-3.53	185.118	15.813	$[M-H]^{-1}$	59.0127/125.0960
10-Oxo-8-decenoic acid	$C_{10} H_{16} O_3$	-3.6	183.102	13.488	$[M-H]-_1$	137.0961/165.0912
16-Hydroxyhexadecanoic acid	$C_{16} H_{32} O_3$	0.47	271.228	29.457	$[M-H]-_1$	225.2221/119.6268/88.0392
4-Methoxycinnamaldehyde	$C_{10} H_{10} O_2$	0.89	163.076	26.249	$[M+H]+_1$	105.0338/
7-Epi-12-hydroxyjasmonic acid	$C_{12} H_{18} O_4$	-1.07	225.113	17.52	$[M-H]-_1$	181.0865/153.0914/137.0962/59.0127
Arginine	$C_6 H_{14} N_4 O_2$	-0.41	175.119	1.703	$[M+H]+_1$	146.9602/118.9651
Azelaic acid	$C_9 H_{16} O_4$	-4.04	187.097	13.211	$[M-H]-_1$	125.0961/169.0862
Mannitol	$C_6 H_{14} O_6$	-3.84	181.071	1.801	$[M-H]-_1$	163.0603/101/0232/89.0232
Gluconolactone	$C_6 H_{10} O_6$	-4.84	159.029	2.536	$[M-H-H_2O]^{-1}$	130.9826/97.0283/
Glycerophosphocholine	$C_8 H_{20} N O_6 P$	-0.13	258.11	1.993	$[M+H]^{+1}$	104.1072/184.0734
Leucylproline	$C_{11} H_{20} N_2 O_3$	0.88	229.155	3.075	$[M+H]+_1$	142.0863
Phaseolic acid	$C_{12} H_{22} O_6$	0.49	261.134	12.457	$[M-H]-_1$	201.1127/225.1130/243.1236/125.0966
Phenylalanine	$C_9 H_{11} N O_2$	0.85	166.086	4.281	$[M+H]+_1$	120.0807/84.9601
Phytosphingosine	$C_{18} H_{39} N O_3$	0.03	318.3	19.834	$[M+H]+_1$	256.2631/102.0916
Quinic acid	$C_7 H_{12} O_6$	-2.65	191.056	2.897	$[M-H]-_1$	102.9476/146.9376
Sphinganine	$C_{18} H_{39} N O_2$	-0.09	302.305	22.212	$[M+H]+_1$	159.1172/137.0967/284.2947
sterol derivative	$C_{28} H_{46} O_2$	-0.15	415.357	30.153	$[M+H]+_1$	396.4201/231.2104/161.1324/95.0858
sterol derivative	$C_{28} H_{44} O_3$	-0.64	429.336	28.901	$[M+H]+_1$	325.3299/371.1609/255.5379
Suberic acid	$C_8 H_{14} O_4$	-4.08	173.081	11.332	$[M-H]-_1$	129.0910/111.0804/155.0704
Trihydroxyoctadecadienoic acid	$C_{18} H_{32} O_5$	0.6	327.218	16.486	$[M-H]-_1$	229.1443/211.1330/171.1017

Table S4: Metabolites identified in *n*-hexane extracts of carrot seeds from the Amatrice (DCA) and Capracotta (DCC) accessions by LC-ESI-MS/MS analysis in positive and negative ionization modes. Metabolite annotation was performed using Compound Discoverer software

Name	Formula	ppm	m/z	RT [min]	Reference Ion	MS/MS
12,13-DHOME	$C_{18}H_{34}O_4$	-0.44	295.228	27.857	$[M-H-H_2O]^{-1}$	277.2173/251.2378/193.1590/83.0490
13-Oxo-9,11-octadecadienoic acid	$C_{18}H_{30}O_3$	0.13	295.227	31.54	$[M+H]^{+1}$	277.2162/81.0703/95.0858
16-Hydroxyhexadecanoic acid	$C_{16}H_{32}O_3$	0.59	271.228	29.471	$[M-H]^{-1}$	225.2222/143.0707/88.0393
1-Phenyl-2-hexanone	$C_{12}H_{16}O$	-0.5	177.127	25.486	$[M+H]^{+1}$	159.1166/149.1323/119.0855/105.0700
2,4-Undecadienal	$C_{11}H_{18}O$	0.36	167.143	31.41	$[M+H]^{+1}$	127.0726/120.0325/1052.0222/84.9601
5-Phenyl-1-pentanol	$C_{11}H_{16}O$	0.63	165.128	29.434	$[M+H]^{+1}$	123.0805/109.0650/95.0859/81.0703
Azelaic acid	$C_9H_{16}O_4$	-3.43	187.097	13.251	$[M-H]^{-1}$	125.0962/169.0865
Camphor	$C_{10}H_{16}O$	0.69	153.128	19.22	$[M+H]^{+1}$	135.1168/107.0857/93.0702
Carvone	$C_{10}H_{14}O$	0.81	151.112	20.065	$[M+H]^{+1}$	109.0649/81.0703/69.0340
Caryophyllene oxide	$C_{15}H_{24}O$	0.47	221.19	29.253	$[M+H]^{+1}$	203.1792/147.1166/135.1167/109.1013/95.0846
Farcarindiol	$C_{17}H_{25}O_2$		261.1854	25.12	$[M+H]^{+1}$	105.0701/81.0703/119.0856/161.1323
Mannitol	$C_6H_{14}O_6$	-3.77	181.071	1.737	$[M-H]^{-1}$	163.0601/136.9088/112.9844/92.9188
Hexadeca-4,7,10,13-tetraenoic acid	$C_{16}H_{24}O_2$	0.12	249.185	27.536	$[M+H]^{+1}$	161.1323/119.0856/105.0701
Isoimperatorin	$C_{16}H_{14}O_4$	0.55	271.097	22.902	$[M+H]^{+1}$	203.0337/175.0392/135.1164/69.0704
MG(18:1/0:0/0:0)	$C_{21}H_{40}O_4$	0.28	357.3	31.781	$[M+H]^{+1}$	339.2878/247.2414/121.10111/95.0859
Oleic acid	$C_{18}H_{34}O_2$	-0.11	281.249	33.103	$[M-H]^{-1}$	263.2369/139.7017
Phytosphingosine	$C_{18}H_{39}NO_3$	-0.19	318.3	19.838	$[M+H]^{+1}$	256.2631/300.2894
sesquiterpene derivative	$C_{15}H_{20}O$	-0.52	217.159	21.821	$[M+H]^{+1}$	159.1167/107.0857/199.1481
Sphinganine	$C_{18}H_{39}NO_2$	-0.37	302.305	22.216	$[M+H]^{+1}$	196.0358/106.0864
Sphingosine	$C_{18}H_{37}NO_2$	-0.42	282.279	26.211	$[M+H-H_2O]^{+1}$	265.2523/247.2414/149.1325/135.1168/83.0859
Tridecanal	$C_{13}H_{26}O$	-3.48	197.19	28.123	$[M-H]^{-1}$	103.9754/153.0911/57.0334

Table S5: Metabolites identified in chloroform extracts of carrot seeds from the Amatrice (DCA) and Capracotta (DCC) accessions by LC-ESI-MS/MS analysis in positive and negative ionization modes. Metabolite annotation was performed using Compound Discoverer software

Name	Formula	DeltaMass [ppm]	m/z	RT [min]	Reference Ion	MS/MS
13-HODE	$C_{18} H_{32} O_3$	-0.58	295.228	28.386	$[M-H]^{-1}$	197.0905/251.2376/151.1117
16-Hydroxyhexadecanoic acid	$C_{16} H_{32} O_3$	0.12	271.228	29.416	$[M-H]^{-1}$	225.2220/253.2171/121.5485
3-(3,4-dimethoxyphenyl)prop-2-enoic acid	$C_{11} H_{12} O_4$	-1	209.081	19.264	$[M+H]^{+1}$	191.0698/163.0748/181.0853
Acetovanillone	$C_9 H_{10} O_3$	-0.94	167.07	13.904	$[M+H]^{+1}$	127.0724/102.9704/84.9000
Azelaic acid	$C_9 H_{16} O_4$	-3.51	187.097	13.267	$[M-H]^{-1}$	125.0967/99.9479
beta-Damascenone	$C_{13} H_{18} O$	-1.73	173.132	20.478	$[M+H-H_2O]^{+1}$	149.1321/135.1166/121.1010
Camphor	$C_{10} H_{16} O$	-1.09	153.127	13.41	$[M+H]^{+1}$	81.0702/93.0700/107.0855/135.1165
Carvone	$C_{10} H_{14} O$	-1.16	151.112	20.097	$[M+H]^{+1}$	133.1010/123.1167/109.0648/93.0701/81.0340
Caryophyllene oxide	$C_{15} H_{24} O$	-0.77	221.19	29.627	$[M+H]^{+1}$	147.1165/161.1321/133.1010
demethylsuberosin	$C_{14} H_{14} O_3$	-0.98	229.087	19.565	$[M-H]^{-1}$	211.1333/167.1433
mannitol	$C_6 H_{14} O_6$	-3.7	181.071	1.742	$[M-H]^{-1}$	163.0603/101.0233/89.0233
Isoferulic acid	$C_{10} H_{10} O_4$	-3.29	193.05	14.919	$[M-H]^{-1}$	149.0598/131.0491
Isoflavone derivative	$C_{20} H_{20} O_4$	-0.98	325.143	22.235	$[M+H]^{+1}$	163.0748/151.0749/137.0593
Isoimperatorin	$C_{16} H_{14} O_4$	-1.33	271.096	22.93	$[M+H]^{+1}$	203.0332/175.0385/69.0703
Linoleic acid	$C_{18} H_{32} O_2$	-0.05	279.233	25.876	$[M-H]^{-1}$	235.1895/136.0516/96.9589
Luteolin	$C_{15} H_{10} O_6$	-0.29	285.04	15.759	$[M-H]^{-1}$	175.039
MG(18:1/0:0/0:0)	$C_{21} H_{40} O_4$	-0.82	357.3	31.805	$[M+H]^{+1}$	247.2412/135.1165/121.1010
MG(18:2/0:0/0:0)	$C_{21} H_{38} O_4$	-0.66	355.284	29.523	$[M+H]^{+1}$	263.2363/245.2256/175.1476
Myristicin	$C_{11} H_{12} O_3$	-1.07	225.112	11.152	$[M+H+MeOH]^{+1}$	193.0854/165.0905
Oleic acid	$C_{18} H_{34} O_2$	-0.89	281.248	33.183	$[M-H]^{-1}$	154.5307/263.2383
Palmitic acid	$C_{16} H_{32} O_2$	-0.41	255.233	32.251	$[M-H]^{-1}$	219.8453/211.1329/121.2729
Phaseolic acid	$C_{12} H_{22} O_6$	0.38	261.134	12.481	$[M-H]^{-1}$	187.0968/125.0960
Phellopterin	$C_{17} H_{16} O_5$	-1	301.107	23.674	$[M+H]^{+1}$	233.0437/218.0204/215.0333/
Phytosphingosine	$C_{18} H_{39} N O_3$	-1.18	318.3	19.474	$[M+H]^{+1}$	256.2627/199.1479/113.0597
Pinocembrin	$C_{15} H_{12} O_4$	-1.15	255.066	18.512	$[M-H]^{-1}$	153.0184/135.0077/119.0491
Retinal	$C_{20} H_{28} O$	-1.17	285.221	29.466	$[M+H]^{+1}$	185.1321/57.0704
Sesquiterpene derivative	$C_{15} H_{20} O$	-1.83	217.158	21.103	$[M+H]^{+1}$	157.1008/199.1477/145.1008
Sphingosine	$C_{18} H_{37} N O_2$	-1.54	300.289	25.682	$[M+H]^{+1}$	281.2121/237.2224/187.9409
Stearic acid	$C_{18} H_{36} O_2$	-0.22	283.264	28.994	$[M-H]^{-1}$	239.1653/149.9037/137.0598

Below **(S10 -S15)** are reported ^{1}H and ^{13}C NMR spectra (1D and 2D) of mannitol (600MHz, D_2O).

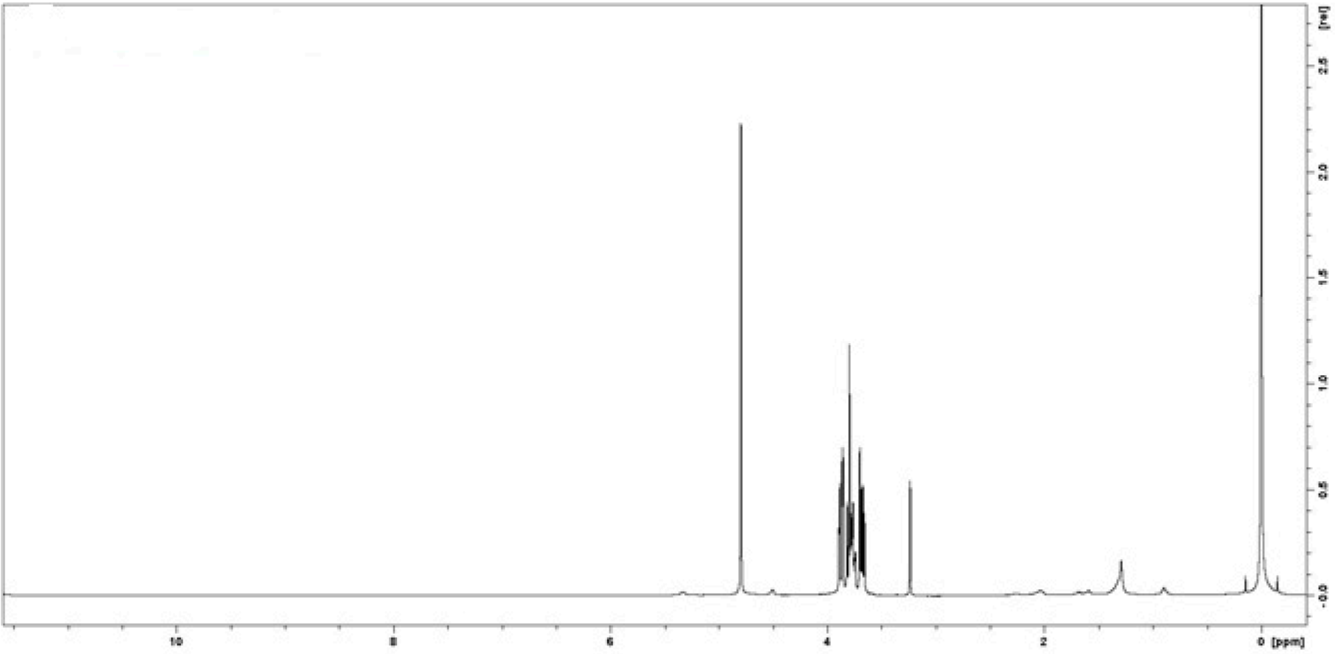

Figure S10: ^{1}H NMR spectrum of mannitol (600MHz, D_2O).

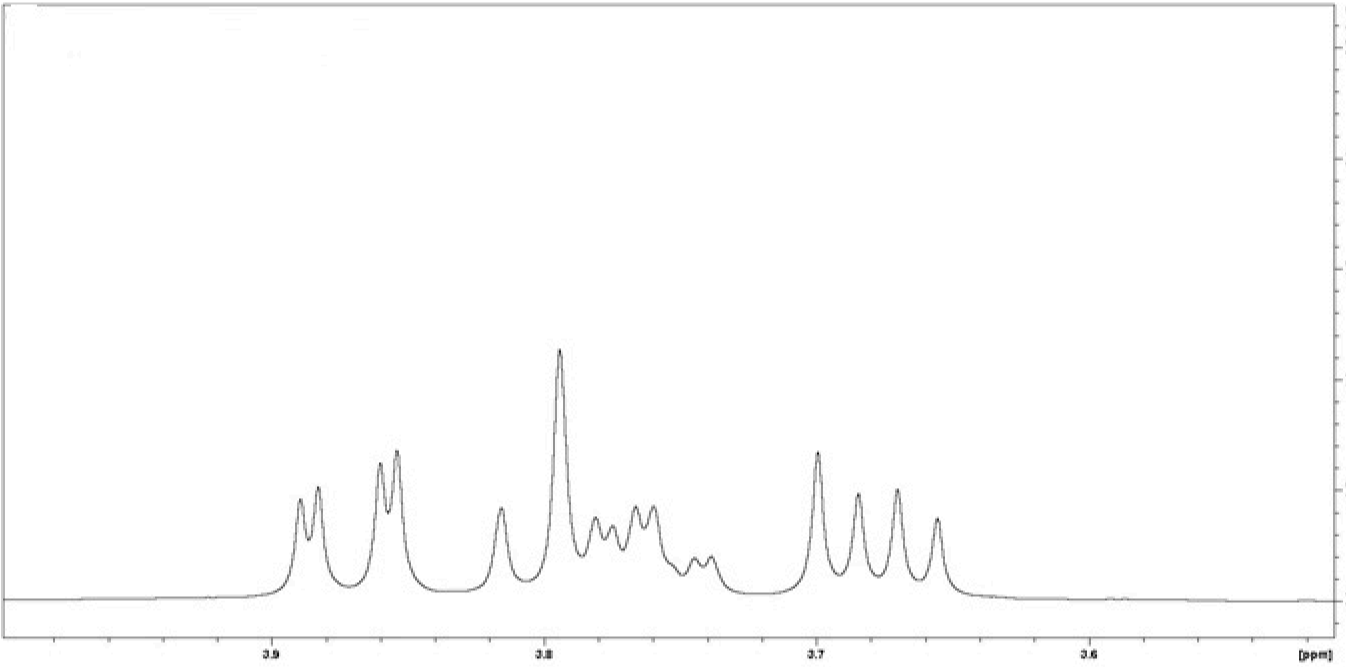

Figure S11: Enlarged region of ^{1}H NMR spectrum of mannitol (600MHz, D_2O).

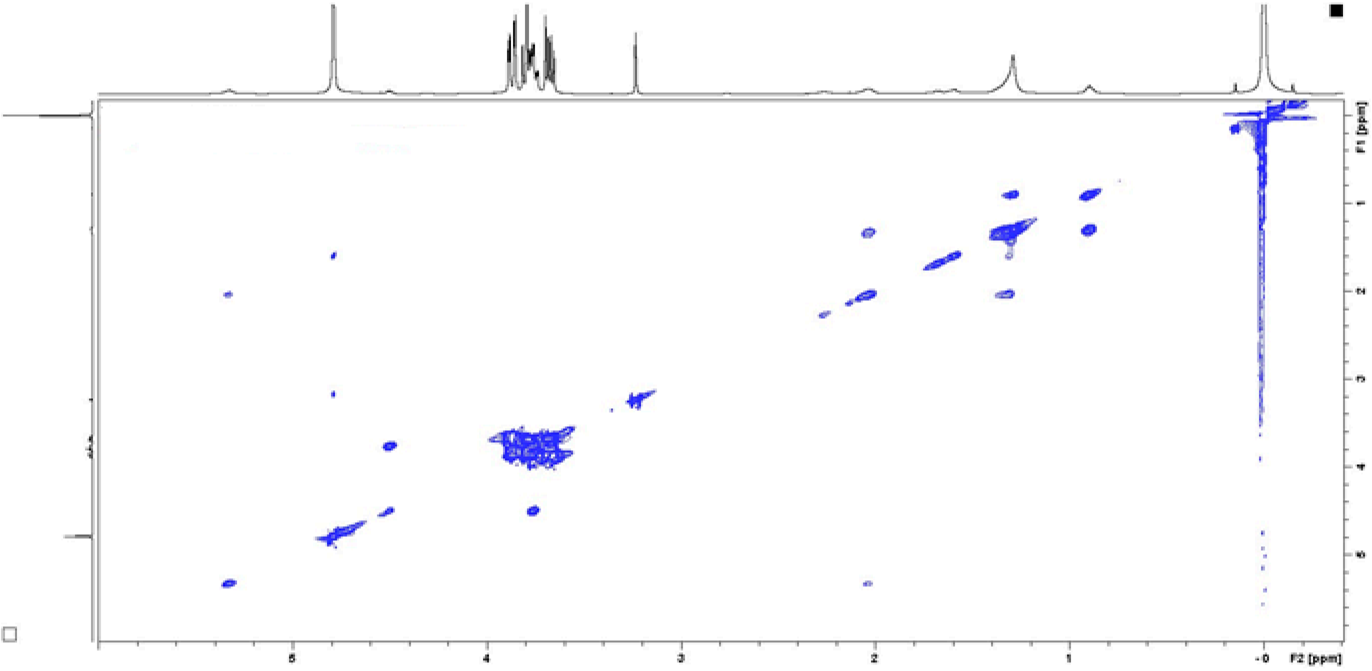

Figure S12: TOCSY NMR spectrum of mannitol (400MHz, D_2O).

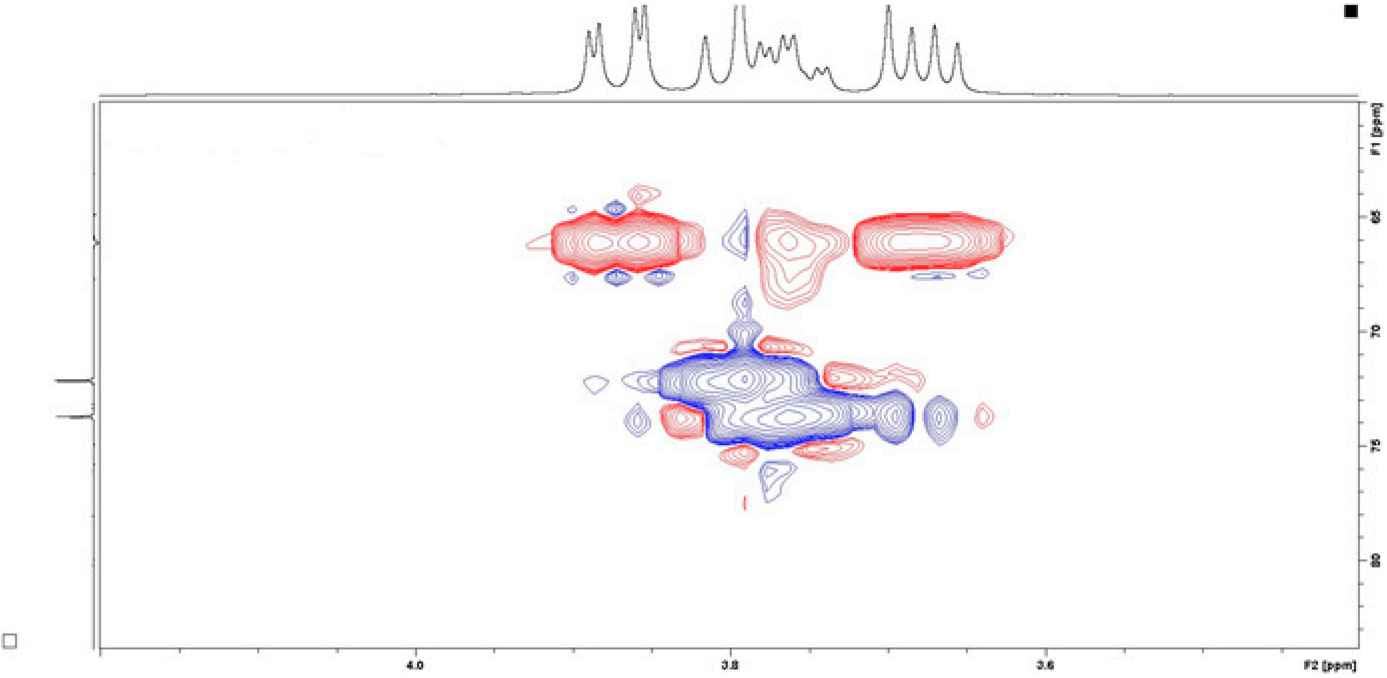

Figure S13: Enlarged region of HSCQ NMR spectrum of mannitol (600MHz, D_2O).

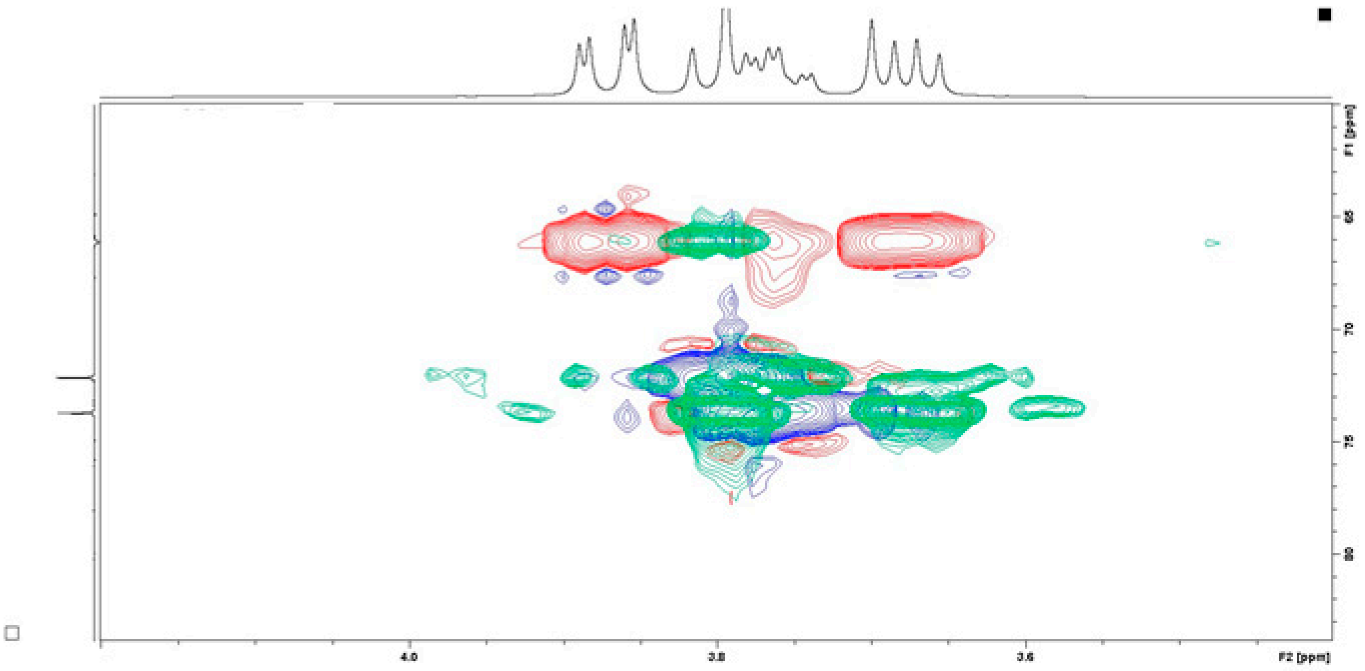

Figure S14: Overlap of an enlarged region of HMBC (green) and HSQC (blu and red) NMR spectra of mannitol (600MHz, D_2O).

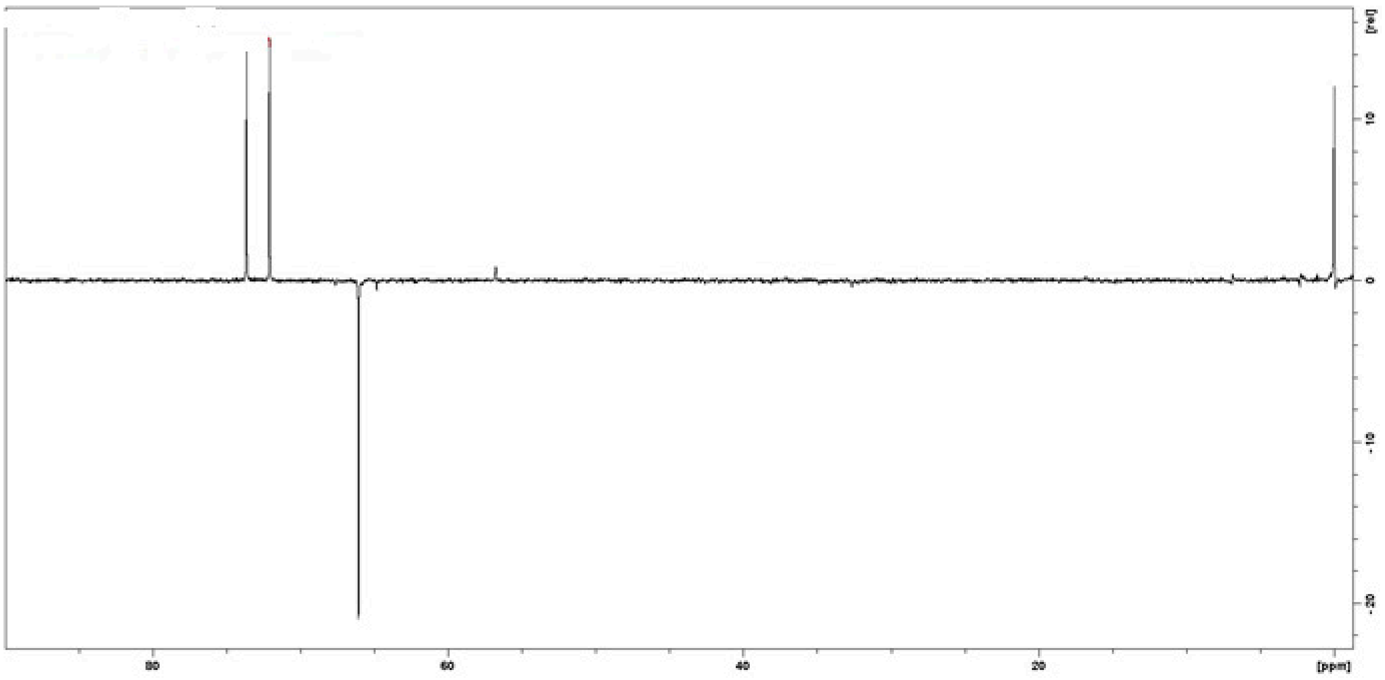

Figure S15: DEPT-135° NMR spectrum of mannitol (400MHz, D_2O).

Table S6 ^{1}H and ^{13}C NMR assignments of Mannitol (D_2O, 600 MHz)

Mannitol	δ_H	δ_C
CH_2 (1-6)	3.68 – 3.87 dd	66.1
CH (2-5)	3.76 m	73.8
CH (3-4)	3.8 d	72.1

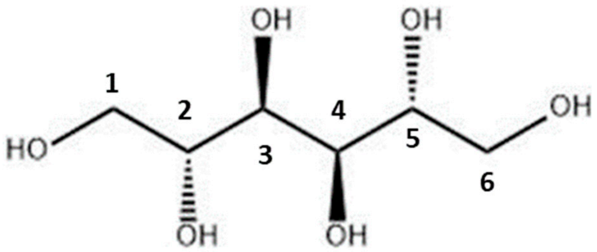

Figure S16: Chemical structure of mannitol.

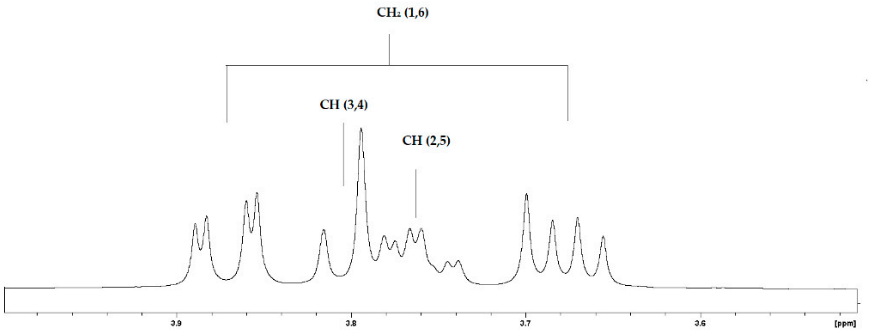

Figure S17: Visual chemical shifts assignment of mannitol referenced to δ_H TMS = 0

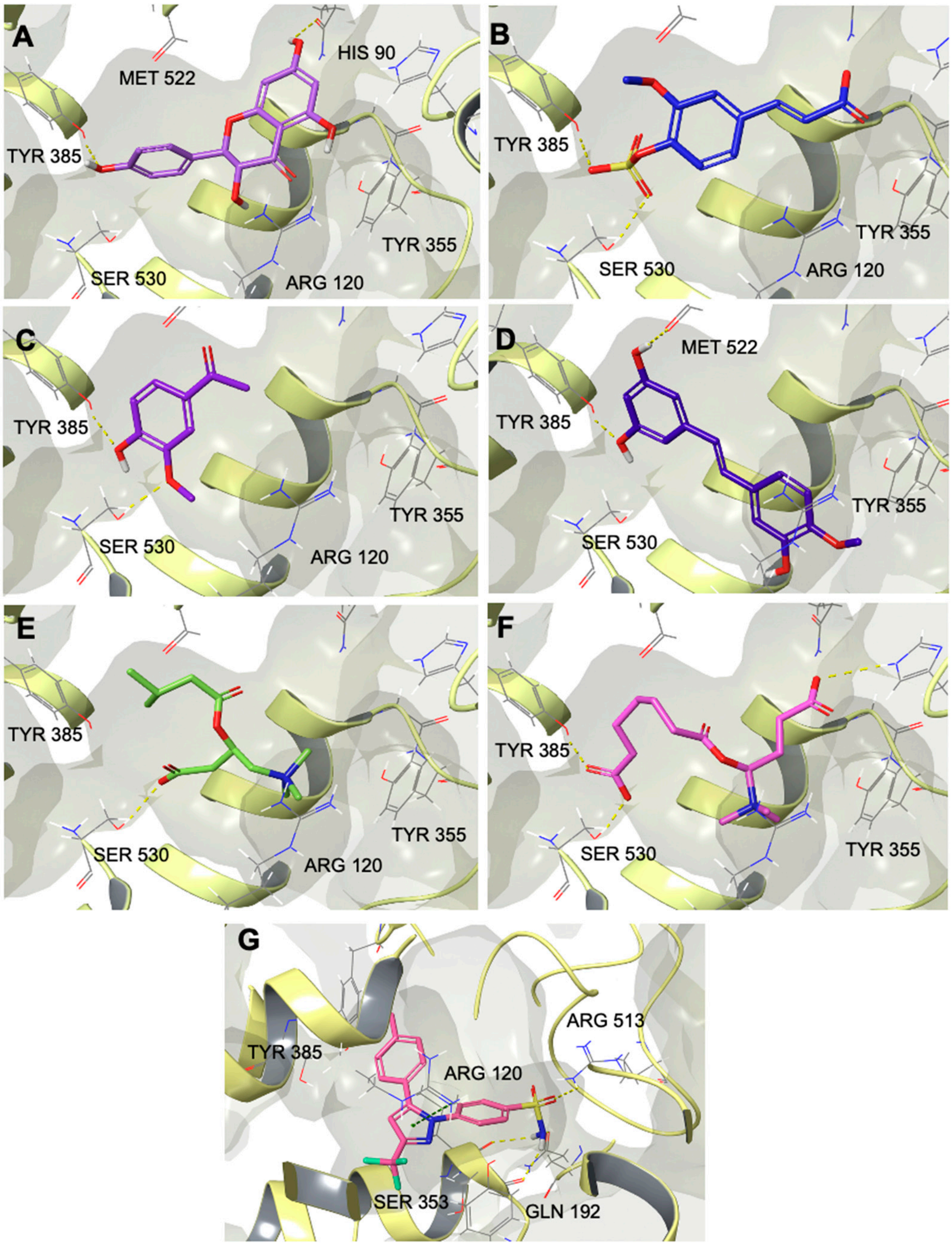

Figure S18. 3D binding mode of A) dihydrokaempferol (colored by atom type: C light purple, O red, polar H white), B) ferulic acid 4-O-sulfate (colored by atom type: C blue, O red, S yellow), C) acetovanillone (colored by atom type: C violet, O red, polar H white), D) rhapontigenin (colored by atom type: C dark violet, O red, polar H white), E) isovalerylcarnitine (colored by atom type: C lime, O red, N blue), F) O-pimeloylcarnitine (colored by atom type: C lilac, O red, N blue) G) celecoxib (colored by atom type: C pink, O red, N blue) in the COX-2 (PDB ID: 5IKQ) catalytic site. Hydrogen bonds and π -cation interactions are represented as dotted yellow and dotted green lines.

Table S7. Docking score reported by the selected compounds against COX-2.

**Compound**	**Docking score (COX-2) (kcal/mol)**
Celecoxib (known inhibitor)	-11.9
14,15-DiHETrE	-11.8

Apigenin	-10.9
12,13-DHOME	-9.5
Dihydrokaempferol	-8.3
Rhapontigenin	-8.3
Tetradecanedioic acid	-7.4
Carvone	-6.9
Acetovanillone	-6.8
Ferulic acid 4-O-sulfate	-6.4
Isovalerylcarnitine	-6.3
Dodecanedioic acid	-5.9
Mannitol	-5.8
O-Pimeloylcarnitine	-5.6
12-Hydroxydodecanoic acid	-5.4
Traumatin	-4.8
10-Hydroxydecanoic acid	-4.2
Sebacic acid	-4.2
9-Oxo-nonanoic acid	-4.1

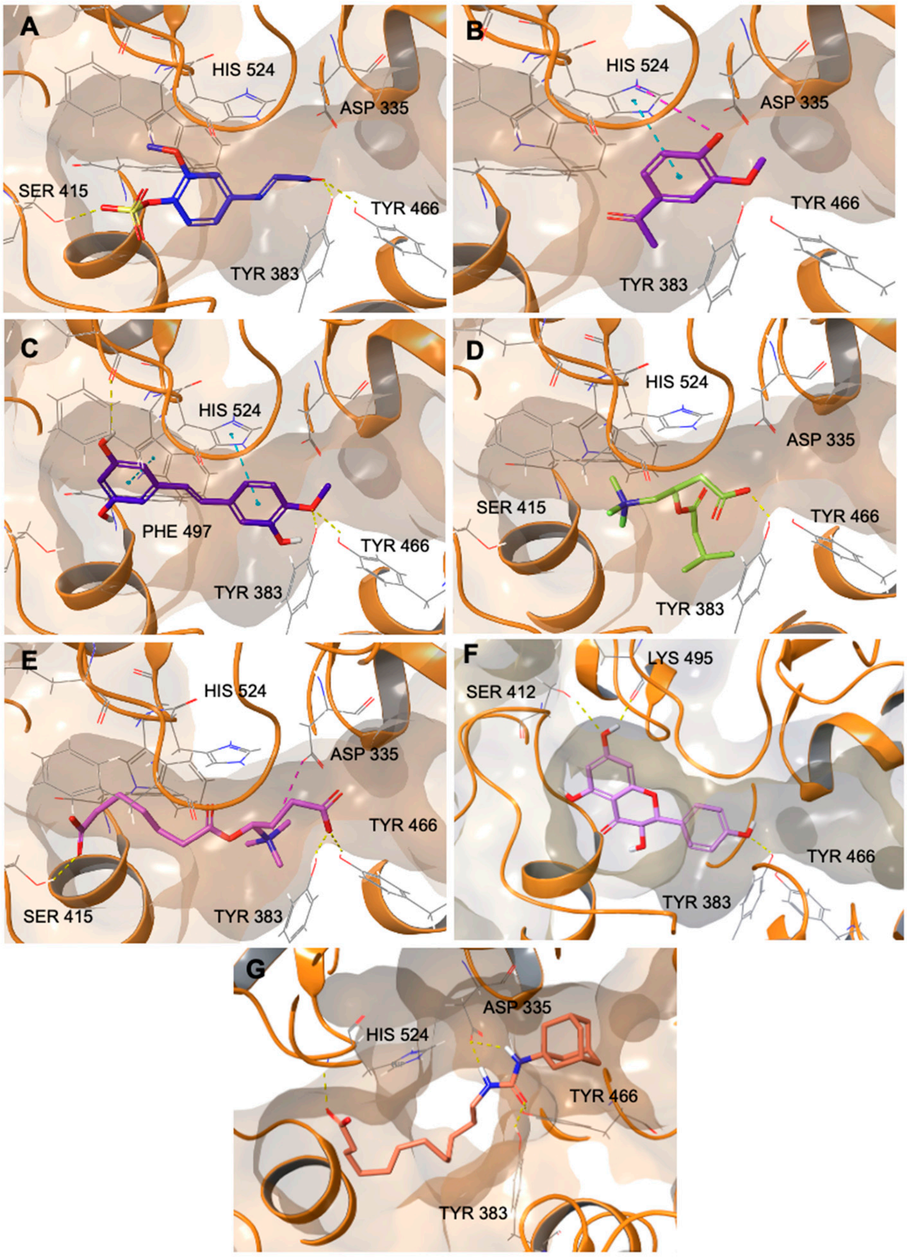

Figure S19. 3D binding mode of A) ferulic acid 4-O-sulfate (colored by atom type: C blue, O red, S yellow), B) acetovanillone (colored by atom type: C violet, O red, polar H white), C) rhapontigenin (colored by atom type: C dark violet, O red, polar H white), D) isovalerylcarnitine (colored by atom type: C lime, O red, N blue), E) O-pimeloylcarnitine (colored by atom type: C lilac, O red, N blue), F) dihydrokaempferol (colored by atom type: C light purple, O red, polar H white), G) AUDA (colored by atom type: C salmon, O red, N blue) in the sEH (PDB ID: 3WKE) catalytic site. Hydrogen bonds, π- π and π-cation interactions are represented as dotted yellow lines, blue lines and purple lines, respectively.

Table S8. Docking score reported by the selected compounds against sEH.

Compound	Docking score (sEH) (kcal/mol)
AUDA (known inibitor)	-13.3
14,15-DiHETrE	-11.1

12,13-DHOME	-10.2
Dihydrokaempferol	-10.0
Apigenin	-9.8
Rhapontigenin	-9.1
Carvone	-7.6
Ferulic acid 4-O-sulfate	-7.4
Tetradecanedioic acid	-6.0
Mannitol	-6.8
Dodecanedioic acid	-5.4
12-Hydroxydodecanoic acid	-5.3
Traumatin	-5.1
Acetovanillone	-4.6
Sebacic acid	-4.5
10-Hydroxydecanoic acid	-4.4
9-Oxo-nonanoic acid	-4.1
O-Pimeloylcarnitine	-3.8
Isovalerylcarnitine	-3.6
